# Synergistic double laser beam-boosted liquid-NIR-SERS for ultralow detection of non-adsorptive polycyclic aromatic hydrocarbons in lake water

**DOI:** 10.1515/nanoph-2022-0010

**Published:** 2022-04-25

**Authors:** Mengya Zhang, Yue Tian, Anxin Jiao, Hui Ma, Chang Wang, Linqi Zheng, Shuang Li, Ming Chen

**Affiliations:** Shandong University, Jinan, China; Shandong Jianzhu University, Jinan, Shandong, China

**Keywords:** double laser excitation, liquid NIR-SERS activity, surface-enhanced Raman scattering spectroscopy, ultra-trace PAHs molecules detection

## Abstract

Ultrasensitive trace-detection of toxic and carcinogenic polycyclic aromatic hydrocarbons (PAHs) can ceaselessly propel the environmental surveillance in aqueous ecosystems. Due to the intrinsic nonadsorptive feature of PAHs, the promising technique of surface-enhanced Raman scattering (SERS) spectroscopy has been restricted to diverse functional ligands-based surface modifications of nano-substrates. However, it is not suitable for practical ultralow liquid analysis. Herein, we propose an extraordinary strategy to boost liquid-near infrared (NIR)-SERS activity of plasmonic Au/Ag nano-urchins (NUs) by introducing extra 808 nm laser-triggered an additional strong electromagnetic enhancement into routine 785 nm laser-Raman system. The synergistic double laser-excited NIR-SERS of colloidal Au/Ag NUs enables the Raman signals of crystal violet to be significantly enhanced, approaching a maximum of ∼34-fold increase than that of traditional bare 785 nm laser-excitation. More importantly, the improved liquid-NIR-SERS enables the *in-situ* detection limit of pyrene molecules in lake water to be achieved at ∼10^−9^ M, which is already better than many previous SERS results based on the complicated functionalized nano-substrates. The established double laser-boosted NIR-SERS can also be easily extended to the simultaneous trace-detection of three PAHs-contaminated mixtures, supporting well distinguishable capability. Undoubtedly, the present work opens a new versatile and innovative avenue for ultrasensitive NIR-SERS monitoring of nonadsorptive toxic pollutants in wastewater.

## 1 Introduction

During the incomplete combustion of coal, petroleum, petrol and other fossil fuels, many discharged byproducts, especially ubiquitous polycyclic aromatic hydrocarbons (PAHs) with two or more benzene rings have inevitably brought serious hazardous effects on living organisms due to their high toxic, carcinogenic, teratogenic, mutagenic and biological accumulative properties [1], [2], [3], [4]. In particular, tremendous damages to human respiratory, urinary, and nervous systems will be caused when long-term exposure to these toxic PAHs contaminants including pyrene, phenanthrene, anthracene, fluorene and their derivatives [2]. Specially, the maximum contaminant level (MCL) of PAHs in drinking water cannot be more than 0.2 ppb (∼10^−9^ M) according to the stipulation of United States Environmental Protection Agency (U.S. EPA) [5, 6]. Therefore, abundant researches have ceaselessly propelled the ultrasensitive detection of PAHs in diverse aqueous ecosystems, aiming to offer reliable and accurate early-assessments of public health and environment protection. Up to now, many commonly used methods with good selectivity and sensitivity have been frequently applied to PAHs analysis, including gas chromatography-mass spectrometry (GC-MS) [7, 8], high-performance liquid chromatography (HPLC) [9, 10], positive-ion atmospheric pressure photo-ionization (APPI) coupled with Fourier transform ion cyclotron resonance (FTICR) [11], condensed phase membrane introduction mass spectrometry with liquid electron ionization (CP-MIMS-LEI) [12], and paper spray ionization mass spectrometry (PSI-MS) [13], etc. However, the tedious sample preparations and pre-concentration steps as well as complicated time/labor-consuming detection processes will create a huge barrier for rapid *in-situ* identification and convenient monitoring of residual trace PAHs in complex wastewater environments.

Alternatively, the surface-enhanced Raman scattering (SERS) spectroscopy derived from strong resonance interaction between incident laser and high-performance nano-substrates can provide molecular vibrational-fingerprints at nanomolar level (10^−9^ M) and possess many capabilities, high specificity and sensitivity as well as simplicity in use [14], [15], [16], [17]. It is generally accepted that the SERS with the increased of Raman signals is mainly dependent on the primary enhanced electromagnetic (EM) field generated by laser-excited strong inelastic scattering on rugged plasmonic (Au, Ag or Cu, etc.) nanomaterials (NMs) with unique local surface plasmonic resonance (LSPR) property [14, 15]. Meanwhile, the minor contribution of the charge-transfer (CT) induced chemical (CM) enhancement has been also confirmed on band-gap semiconductor nano-substrates [16]. Up to now, the emerging SERS has been extensively applied to many cutting-edge fields; however some special non-adsorptive probe molecules with intrinsic nonpolar nature have poor accessibility to the plasmonic metallic surfaces, which cannot be effectively enhanced through the above-mentioned EM or CM enhancement. Unfortunately, the toxic PAHs with multiplex adjoined aromatic-ring structures, as representative non-adsorptive molecules, do not have any metal-affinity functional groups. It prevents effective chemisorption of PAHs on plasmonic surfaces, which lead to PAHs being “inactive” or even “invisible” in most cases for traditional SERS [1, 4, 5, 18], [19], [20], [21], [22]. In order to yield strong Raman signals of PAHs and obtain desirable SERS analysis, many efforts have been dedicated to functionalize the as-prepared nano-substrates by surface chemical modifications, such as Au nanoparticles (NPs) decorated glycidyl methacrylate-ethylene dimethacrylate (GMA-EDMA) [23], ammonium pillar [5]arene-stabilized Au NPs [19, 24], poly-*N*-isopropylacrylamide (pNIPAM)-coated Au nanostars [22], thiol-modified Fe_3_O_4_@Ag [18], mono-6-thio-β-cyclodextrin (HS-β-CD)-captured Au NPs [4], polymer chitosan (CS) Ag NPs [1] Cu_3_(BTC)_2_ (BTC = 1,3,5-benzenetricarboxylic acid)@Ag NPs [25], etc. Although the mentioned plentiful chemical modifications can enhance SERS activity of PAHs, there are still two urgent issues should be taken into consideration toward widespread applications. Firstly, these modified hybridizations are solely restricted to the solid phase-detection of dried PAHs adsorbed on the functionalized nano-substrates. It is not suitable for ultralow liquid-SERS analysis in practical aqueous conditions, due to the weaker chemisorbed capacity of functional ligands bonded with metallic surface and probe PAHs in solution. On the other hand, during chemical functionalization of plasmonic nanocrystals, the added polymer organic molecules that served as anchors allowing the PAHs to close to plasmonic surfaces would result in secondary pollution in practical wastewater surveillance. Moreover, they will also occupy the SERS active sites on nano-substrates, inhibiting the formation of “hot spots” and interfering the Raman signals of probe molecules [20, 26]. Therefore, some unusual methods without the aid of polymer functional ligands have been also customized to improve SERS activity of PAHs via electrochemical cyclic voltammetry treatment of Ag nanowires [27], dispersive liquid–liquid, solid phase or *in-situ* surface microextractions [28], [29], [30], molecularly imprinted polymers (MIPs) with Au NPs assemblies [31], and glass fiber paper combined Ag NPs [32], etc. Despite these emerging strategies can be applied to the *in-situ* liquid-SERS detection of PAHs, the rigorous pretreatments of the complicated nano-substrates will be also inconvenient for rapid replicable and reproducible SERS analysis. Up to now, how to realize the accessible, robust, versatile and stable *in-situ* liquid-SERS monitoring of ultra-trace PAHs still face a huge challenge for wastewater surveillance in real-world scenarios.

**Figure 1:**
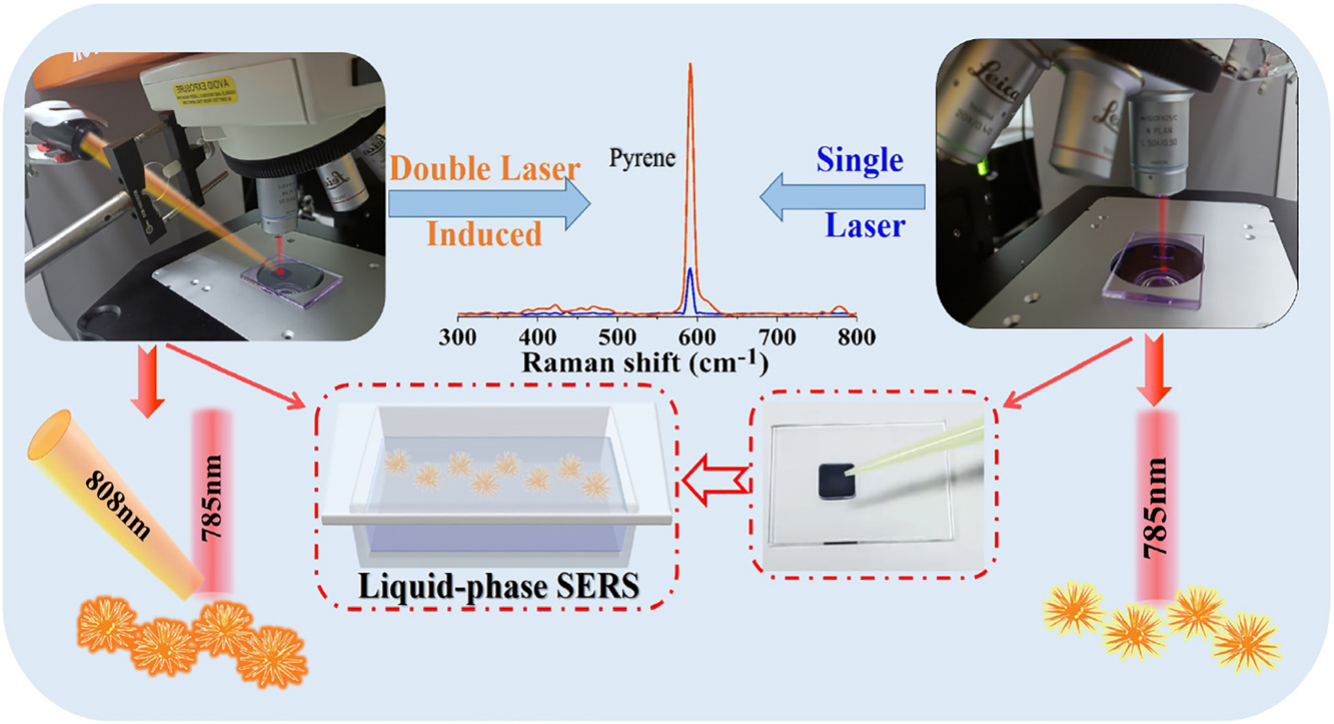
The schematic diagram of double laser beam-excited equipment for further boosting liquid-NIR-SERS enhancement of non-adsorptive toxic PAHs in solution.

Herein, we report an extraordinarily versatile strategy for realizing *in-situ* ultrasensitive liquid-detection of trace PAHs in real-word lake water based on synergistic double laser beam-boosted near infrared (NIR)-SERS. It simultaneously possesses many distinctive features: (1) superior to traditional SERS analysis via ultraviolet (UV) or visible light (<700 nm) excitation, the NIR-SERS with high liquid-penetrating capability is suitable for molecular dynamic liquid-monitoring/sensing of diverse analytes in complex solutions [33], [34], [35], [36], [37], [38], [39]. It can reliably unveil molecular structures owing to the lower photonic energy induced minor damage to samples, which will also significantly weaken many unwanted fluorescence background/interferences that originated from short wavelength excitation [40, 41]; (2) as for traditional NIR-SERS, the main drawback is the insufficient Raman enhancement due to the inadequate efficiency of intrinsic low photon absorption-conversion at long-wavelength region. In this work, the introduction of extra 808 nm laser beam-trigged an additional strong EM field into routine 785 nm laser-Raman equipment can significantly further promoting liquid-NIR-SERS activity of as-prepared colloidal plasmonic NMs, as clearly illustrated in Figure 1; (3) the extremely easy-to-use of double laser beam-boosted liquid NIR-SERS with simplicity, rapidity and ultra-sensitivity can overcome the previous difficulties in complicated surface functionalization of SERS-active nano-substrates by either chemical or physical modifications. Taken together, the double laser-boosted NIR-SERS performed on as-prepared colloidal Au/Ag nano-urchins (NUs) in this work enables the Raman signals of crystal violet (CV) to be significantly enhanced in solution, reaching a maximum of approximate ∼34-fold higher than that of bare 785 nm laser-excitation. More importantly, the liquid-NIR-SERS of pyrene molecules that regarded as a biological exposure index (BEI) for assessment of exposure to PAHs [30], can be also clearly distinguishable even the concentration as low as 1 × 10^−9^ M in practical lake water. The liquid-NIR-SERS detection limit obtained by synergistic double laser excitation is already comparable to or even better than many previous reports via complex functionalized SERS nano-substrates by tedious chemical or physical modifications [1, 5, 18], [19], [20], [21, 23], [24], [25, 27, 28, 31, 32]. Additionally, we also verify the versatility, high sensitivity, and uniformity of this novel double laser-trigged NIR-SERS. Therefore, the present work can avoid previous tedious chemical and/or physical modifications of plasmonic surfaces and provide a new approach for ultrasensitive monitoring of nonadsorptive toxic PAHs pollutants in wastewater.

## 2 Experimental setup

### 2.1 Chemicals and reagents

Silver nitrate (AgNO_3_, 99.85%) and chloroauric acid (HAuCl_4_) were purchased from China Sinopharm International (Shanghai) Co., Ltd. Ascorbic acid (AA, 99%) and ethanol reagents were purchased from Macklin. All the probe molecules (crystal violet, tetracycline (TC), pyrene, anthracene and nitropyrene) were purchased from Aladdin Chemistry Co., Ltd (Shanghai, China). In addition, cetyltrimethylammonium bromide (CTAB, >98%) and polyvinylpyrrolidone (PVP, Mw = 40,000) were purchased from TCI (Shanghai). The custom quartz tank (10 × 10 × 1 mm) was made in Lianyungang. All reagents were of analytical grade and used directly without further treatment. Deionized (DI) water used in the fabrication and measurement was prepared using a Millipore purification system (18.2 MΩ cm).

### 2.2 Synthesis of Au nanorods

The Au nanorods (NRs) were fabricated by a seed-mediated growth method [42, 43]. A 100 mL mixed aqueous solution containing 50 mL CTAB (0.2 M) and 50 mL HAuCl_4_ (0.5 mM) was added in a glass dish. Then added 6 mL of ice-cold NaBH_4_ (0.01 M) to the above solution with vigorous stirring for 3 min, and the solution will immediately turn brown and yellow. The prepared mixed solution was placed in a water bath at 30 °C for 2 h for subsequent use as a seed solution. The prepared mixed solution was immersed in a 30 °C water bath for 2 h to obtain gold seeds with a diameter of 2–3 nm. In a clean beaker, 50 mL CTAB (0.2 M), 50 mL HAuCl_4_ (1 mM), 330 μL AgNO_3_ (0.04 mM), 600 μL AA (0.1 M) and 150 μL of seeds solution were successively added to prepare ∼100 mL growth solution. Then the growth solution was placed in a water bath maintained at 30 °C for 6 h to obtain Au NRs with average diameter and radius of 46 and 12 nm, respectively. The as-synthesized Au NRs were centrifuged at 15,645×*g* rcf for 15 min, the precipitate was then re-suspended in DI water and centrifuged at 15,645×*g* rcf for 15 min. Repeat cleaning for three times to obtain 1 mg/mL Au NRs.

### 2.3 Synthesis of Au/Ag NUs

The preparation of Au/Ag NUs based on liquid laser irradiation is very similar to previous works [44, 45]. The Au/Ag NPs were obtained via laser ablation and irradiation process by a Q-switched Nd-YAG (yttrium aluminum garnet) laser (Quanta Ray, Spectra Physics) beam. A well-polished gold target was first placed at the bottom of a rotating glass dish (400 rpm) containing 5 mm depth of liquid solution (0.2 M AgNO_3_, 0.08 M PVP, and 20 mL DI water). Firstly, a pulsed laser with a wavelength of 1064 nm, a pulse width of about 10 ns, energy of about 250 mJ and a repetition rate of 10 Hz was used to ablate Au target in solution for 10 min. Then, the gold target was removed from the solution, and a pulse laser with a wavelength of 532 nm, a pulse width of about 6 ns, energy of about 350 mJ and a repetition rate of 10 Hz was used to irradiate the above solutions for 20 min. After these two steps, we synthesized Au/Ag NPs with the average size of 5 nm, fully washed Au/Ag NPs with DI water, centrifuged at 22,858×*g* rcf, and dispersed the products into DI water to obtain 0.1 M Au/Ag NPs. 50 mL HAuCl_4_ (0.5 mM) and 16.6 mL AA (0.08 M) were added to 33.4 mL Au/Ag NPs (0.1 M), then stood for 1 min to obtain 100 mL Au/Ag NUs with diameters around 150 nm. The as-synthesized Au/Ag NUs were centrifuged at 4450×*g* rcf for 15 min, the precipitate was then re-suspended in DI water and centrifuged at 4450×*g* rcf for 15 min. Repeat cleaning for three times to obtain 1 mg/mL Au/Ag NUs.

### 2.4 Materials characterization

The obtained precipitates were dropped on a copper mesh and dried in an oven for observation via transmission electron microscopy (JEOL-JEM-2100F, 200 kV, 400,000×). The absorption spectra were carried out via UV–Vis–IR spectrometer (Shimadzu, UV-1800, with a resolution of 1 nm). All the Raman signals were collected by a confocal microprobe Raman spectrometer (Renishaw Raman spectroscopy, with a spectral resolution of 1 cm^−1^).

### 2.5 Liquid-NIR-SERS testing

All liquid-NIR-SERS signals were collected by a confocal microprobe Raman spectrometer (Renishaw Raman spectroscopy) with a 50 × objective. For SERS detection of CV molecules, 100 μL Au NRs (1 mg/mL) or Au/Ag NUs (1 mg/mL) solution and 100 μL of CV molecules (2 × 10^−5^ M CV) solution were added to a 2 mL centrifuge tube, and the concentration of nano-substrates was fixed at 1 mg/mL in each subsequent experiment. Then the above solution was mixed uniformly by ultrasonic for 2–3 min. In this way, Raman detection samples of 10^−5^ M CV molecules could be simply made and the similar process was carried out for the other probe molecules under different concentrations (10^−5^–10^−12^ M). As for NIR-SERS detection of PAHs molecules, the probe molecules were firstly dissolved in ethanol to obtain a high concentration solution, which will be separately diluted with DI water to prepare probe molecules with different concentrations of 10^−5^–10^−10^ M. The sample was placed in a custom closed quartz tank (10 × 10 × 1 mm) for Raman test. The NIR-SERS spectra of CV and TC molecules were recorded at room temperature using a 785 nm laser with an output power of 4.5 mW and the acquisition time used for one spectrum was 1 s. The NIR-SERS spectra of PAHs molecules used a 785 nm laser with an output power of 4.5 mW, the acquisition time used for one spectrum was 10 s, since we have found that the branched microstructures of Au/Ag NUs would be damaged by using the high laser power (∼5.0 mW, the acquisition time of 1 s) in this work. All Raman spectra were obtained using the InVia spectrometers and processed (smoothed and baseline corrected) using Wire 5.4 software (Renishaw). The extra 808 nm laser induced Raman background noises can be easily removed by using the baseline-corrected function in Wire 5.4 software (Figure S1). Moreover, each Raman result was the average value of the Raman tests at 10 different positions of the sample in order to ensure the test accuracy. Before the SERS analyses, the optimal detection location along the vertical direction were selected based on the comparative Raman signal results at different depths below the solution surface. Then the confocal region of the two laser beams was determined by an ingenious method. The *XY* two-way adjustable precision optical displacement platform was added at the bottom of the 808 nm laser (Changchun Laser Optoelectronics Technology Co., Ltd., with the output power ranges of 0–1080 mW), and the focusing spot position of the 808 nm laser was adjusted by adjusting the platform, so as to realize the effect of the 785 nm laser moving to the center of the 808 nm laser beam. The confocal position of the double beam was determined by comparing the Raman signal intensities at different points. First, the platform was adjusted along the *X*-direction to find the strongest signal point *ⅲ* (0, 0) and determine the coordinate region of the confocal on the *X*-axis. Then, the above operations were performed on the *Y*-axis to finally determine the confocal region *ⅶ* (0, 100) of the two lasers.

### 2.6 Liquid-NIR-SERS detection of pyrene in lake water

The lake water was collected from a lake located in the outskirts of Jinan. Before formal use, the samples were firstly filtered by a 0.22 μm filter (Whatman) and then centrifuged at 22,858×*g* rcf to further remove sediment from the lake water. Pyrene-spiked solutions (concentrations ranging from 10^−5^ to 10^−10^ M) were all prepared by using the as-pre-treated lake water. The rest of the procedure was analogous as the above experimental steps.

### 2.7 Finite-difference time domain (FDTD) calculation

The FDTD (package of Comsol Multiphysics 5.3a) method was used to perform the simulations about electromagnetic response of single Au NR and Au/Ag NU. We investigated the relative electrical field intensities at the surfaces of individual Au NR and Au/Ag NU. The Au NR was modeled as a cylinder with two spheres, and Au/Ag NU was modeled as a sphere plus with tens of cylinders. In the simulations, the refractive index of surrounding medium was set as 1.33. Then the excitation wavelength in FDTD simulations was set at 785 and 808 nm according to the experiments.

## 3 Results and discussion

**Figure 2:**
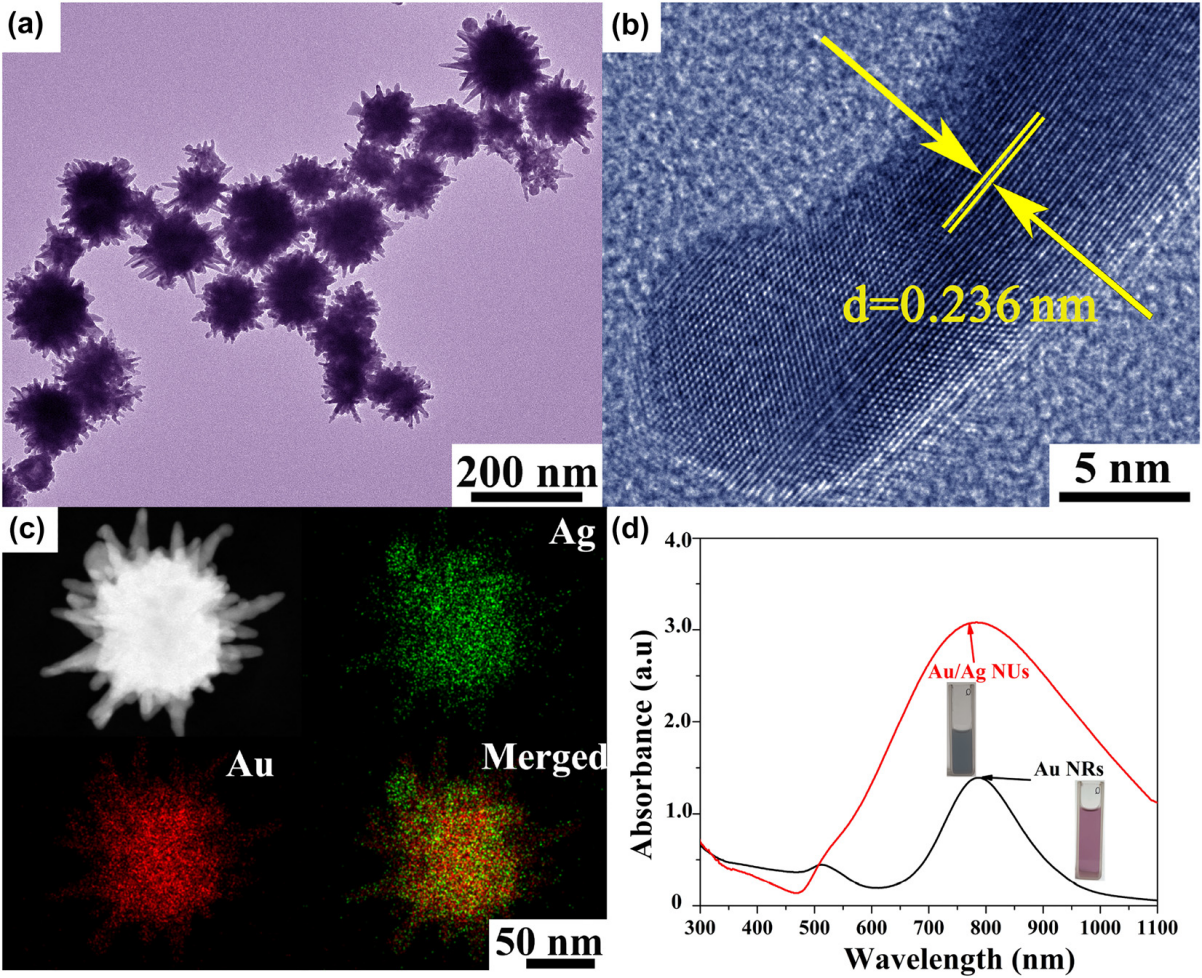
The microstructure of nanoproducts. (a) The TEM image and (b) representative HRTEM image of Au/Ag NUs. (c) The typical enlarged TEM image and the element mapping images of an individual Au/Ag NU. (d) The absorption spectra of colloidal Au/Ag NUs and Au NRs with the concentration of 0.01 mg/mL in each one.

Initially, the anisotropic Au/Ag NUs and Au NRs samples were adopted as two different typical NIR-SERS nano-substrates in this work. The morphology of as-prepared Au/Ag NUs was illustrated by the transmission electron microscopy (TEM), as shown in Figure 2(a). Clearly, it can be seen that numerous mono-dispersed Au/Ag NUs possess abundant and obvious elongated branches. Then, the high-resolution TEM (HRTEM) image in Figure 2(b) provides the detailed structure of the closer view of an isolated nano-urchin with well crystal structure. Correspondingly, the parallel lattice fringe with a *d*-spacing of 0.236 nm is located between theoretical values of (111) lattice plane of Ag (0.238 nm) and Au (0.232 nm), supporting the formation of bimetallic Au/Ag NUs. To further verify the bimetallic nature of Au/Ag NUs, we investigated the elemental mapping images of an individual nanostructure. As displayed in Figure 2(c), the Au and Ag elements are homogeneously distributed on the whole body and the relative ratio of Au and Ag elements is calculated about 88:12, which further confirms the alloying state of the synthesized Au/Ag NUs. Additionally, the detailed analyses of well-defined rod-like structures of Au NRs are then illustrated in Figure S2. Indeed, the choice of as-prepared plasmonic nanostructures is highly related to their LSPR positions, which should be sensitive to the inherent NIR 785 nm wavelength excitation in SERS apparatus. The collective oscillation of plasmonic conduction electrons at resonance frequency in response to incoming laser wavelength will play an important role in high-performance SERS [46], [47], [48]. In this work, as shown in Figure 2(d), the LSPR positions of as-prepared colloidal Au/Ag NUs and Au NRs are located at 781 and 787 nm, respectively, which well match the excitation light source equipped with Raman spectrometer. According to the experimental absorption spectrum, the peak intensity of Au/Ag NUs is approximate 2.3 times higher than that of Au NRs at 785 nm, implying the higher extinction coefficient of Au/Ag NUs. The two typical plasmonic nano-substrates are then selected in our experimental liquid-NIR-SERS tests.

**Figure 3:**
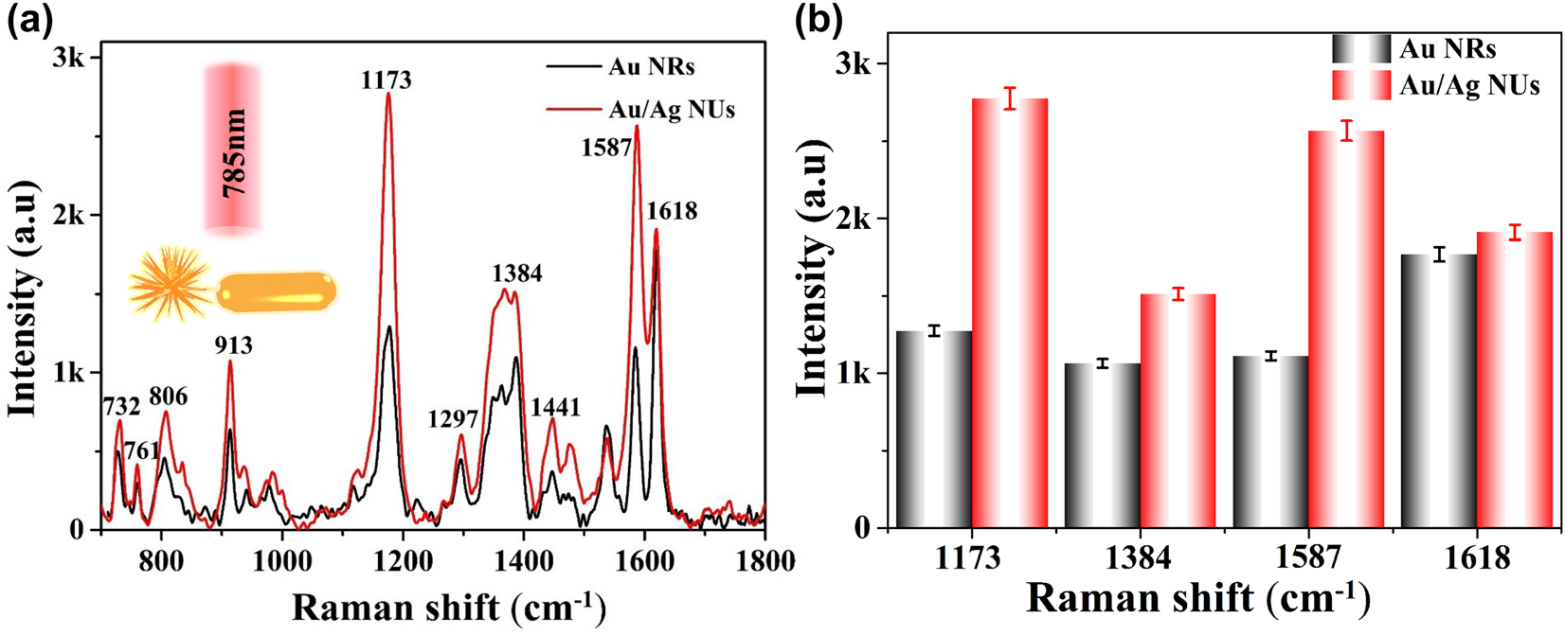
SERS performances of Au/Ag NUs and Au NRs in the single laser system. (a) Raman spectra of 10^−5^ M CV molecules separately performed in the presence of Au/Ag NUs and Au NRs in solution. (b) The comparative results of Raman intensities of CV molecules on two nano-substrates (each error bar indicates the standard deviation of 10 different spots).

The single 785 nm laser induced liquid-NIR-SERS activities of Au NRs and Au/Ag NUs were evaluated by using CV aromatic dye molecules that can effectively connect with plasmonic NMs via strong π–π interactions [49, 50]. After the Au NRs or Au/Ag NUs solutions were uniformly mixed with the probe molecule solutions, the chosen vertical location of each Raman test in this paper was fixed at about 100 nm below the liquid surface, based on the variation of Raman peak intensities via the different depths along the *Z*-axis direction as shown in Figure S3. Based on the 10^−5^ M CV molecules absorbed on the two types of nano-substrates on solution, the liquid-NIR-SERS spectra without and with baseline subtractions were separately shown in Figures S4 and 3(a). As shown in Figure 3(a), the dominating characteristic bands of CV molecules are all clearly detected in Raman spectra, providing much enriched “molecular fingerprint” information. In detail, the Raman peaks at 1618, 1587, 1441 and 1297 cm^−1^ can be attributed to ring C–C stretching vibration; the peak at 1384 cm^−1^ is related to *N*-phenyl stretching; the peak at 913 cm^−1^ should be originated from ring skeletal vibration of radical orientations; the peaks at 1173, 806, 761 and 732 cm^−1^ are corresponded to ring C–H bends [51], [52], [53], [54]. Compared with the traditional solid-SERS measurements, the NIR-SERS signals of probe molecules are relative weaker in solution, owing to the lower molecular concentration in solution and the liquid-fluid medium [55]. As displayed in Figure 3(a), it can be seen that the Au/Ag NUs provide a higher NIR-SERS activity than that of Au NRs. The comparative results of different Raman peak intensities separately originated from Au/Ag NUs and Au NRs are summarized in Figure 3(b). It indicates that the dominating characteristic bands of CV molecules in colloidal Au/Ag NUs are all higher than those of Au NRs. Compared to Au NRs with smooth surfaces, the obtained Au/Ag NUs with plentiful surface nano-antennas can exhibit stronger electromagnetic field located around multiple tip branches due to the generation of more SERS hot spots. As displayed in Figure S5, the relative electrical field intensities distributions reveal that the Au/Ag NUs provide significantly enhanced intense electric fields than that of Au NRs. Therefore, the FDTD simulations suggest that the Au/Ag NUs can provide an enhanced EM field, facilitating higher liquid-NIR-SERS activity in this paper.

**Figure 4:**
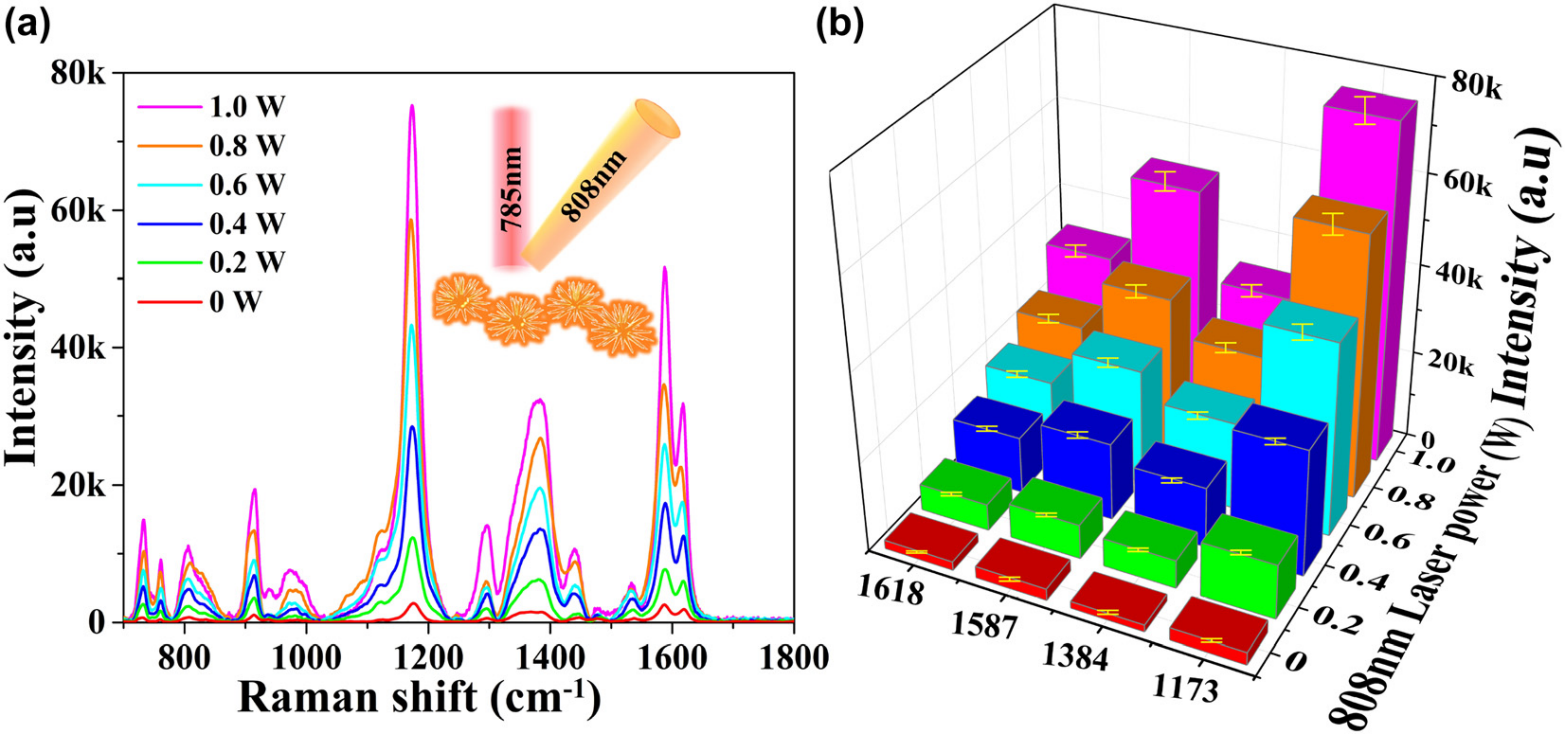
The SERS performances of Au/Ag NUs in the double laser-induced system. (a) Raman signals of CV (10^−5^ M) in Au/Ag NUs solution with different extra laser powers. (b) The variation trends of the main four Raman peak intensities of CV molecules (10^−5^ M) versus the extra 808 nm laser powers (0–1.0 W) (each error bar indicates the standard deviation of 10 different spots).

Different from traditional NIR-SERS by single 785 nm laser excitation, then another 808 nm continuous laser beam was introduced into the routine Raman tests. The EM field around the hot spots of plasmonic nano-architectures can be further boosted by the secondary laser excitation, which is expected to optimize the liquid-NIR-SERS activity. As for double laser-excited NIR-SERS, the optimal confocal region of extra 808 nm laser beam with respect to the routine 785 nm focused point should be illustrated in this work, which is essential to improve NIR-SERS performance. The procedure for determining the optimal confocal region of the two laser beams is described in Figure S6(a). After the colloidal Au/Ag NUs uniformly mixed with CV solution, the Raman spectra and corresponding peak intensities of CV molecules versus the relative positions (*X*-axis and *Y*-axis) of extra 808 nm (1.0 W) and intrinsic 785 nm were separately illustrated in Figure S6(b–f). It can be seen that the Raman signals of CV molecules derived from optimal coordinate point are much higher than that at other deviation points with different offsets. The obtained spatial location with higher Raman signals should be served as double-laser confocal region, which will be fixed in the subsequent experiments. On the other hand, the effect of extra laser power on NIR-SERS enhancement is evaluated in this work. The Raman signals of CV (10^−5^ M) in Au/Ag NUs solution versus different extra 808 nm laser powers (0, 0.2, 0.4, 0.6, 0.8 and 1.0 W) performed in double laser-excited NIR-SERS system are separately shown in Figure 4(a). Meanwhile, the variations of the main four Raman peak intensities of CV versus the extra 808 nm laser power (0–1.0 W) are shown in Figure 4(b). It reveals that the corresponding Raman peak intensities of CV molecules can be effectively promoted by increasing the 808 nm laser power (0–1.0 W). For instance, the Raman peak at 1173 cm^−1^ increases from ∼2803 a.u for traditional single SERS to ∼43,300 a.u for 0.6 W extra laser irradiation, and then to a maximum of ∼75,793 a.u at 1.0 W condition. Clearly, the double laser-induced NIR-SERS system with 1.0 W extra 808 nm laser excitation gives rise to the strongest Raman signals, approaching about 27 times higher than that of original single 785 nm laser beam. Meanwhile, the stability of this NIR-SERS system in the presence of Au/Ag NUs in solution has been confirmed by the Raman tests during double laser irradiation of 60 min at the same point, as shown in Figure S7. On the other hand, we also found that the excess extra laser power (>1.0 W in this experiment) will also result in some uncontrollable damages of plasmonic nanostructures, which is not conducive for the practical NIR-SERS applications. As shown in Figure 4(b), the variation trends of these four groups of Raman peak intensities exhibit very similar behaviors, providing a much higher SERS activity under double laser-induced NIR-SERS in the presence of 1.0 W extra 808 nm laser excitation. As summarized in Table S1, the enhancement degree of each characteristic peak in the double laser-excited NIR-SERS is different, and its maximum value is calculated to be approximate ∼34-fold that of traditional single wavelength (785 nm) excitation. The apparent enhancement of double laser-induced liquid NIR-SERS activity should be attributed to the additional EM field generated by resonance excitation of extra 808 nm laser, which can be effectively raised by increasing laser power in the range of 0–1.0 W. Furthermore, we carried out the point-to-point statistics of the variations of Raman peak at 1173 cm^−1^ in the single laser system and double laser system by elevating the power of the 785 nm laser excitation source. As shown in Figure S8, the increasing trend of Raman peak intensity in double laser system is higher than that in single laser system. It implies that the synergistic coupling effect between the additional EM field via extra 808 nm laser and the intrinsic EM field via original 785 nm laser can play an important role in the double laser-induced NIR-SERS. Moreover, the colloidal Au/Ag NUs are replaced by Au NRs to verify the universal adaptation of double laser induced NIR-SERS enhancement. As shown in Figure S9, the liquid NIR-SERS activity based on Au NRs can be also enhanced by the double laser-excited strategy, implying the versatility of this innovative approach.

**Figure 5:**
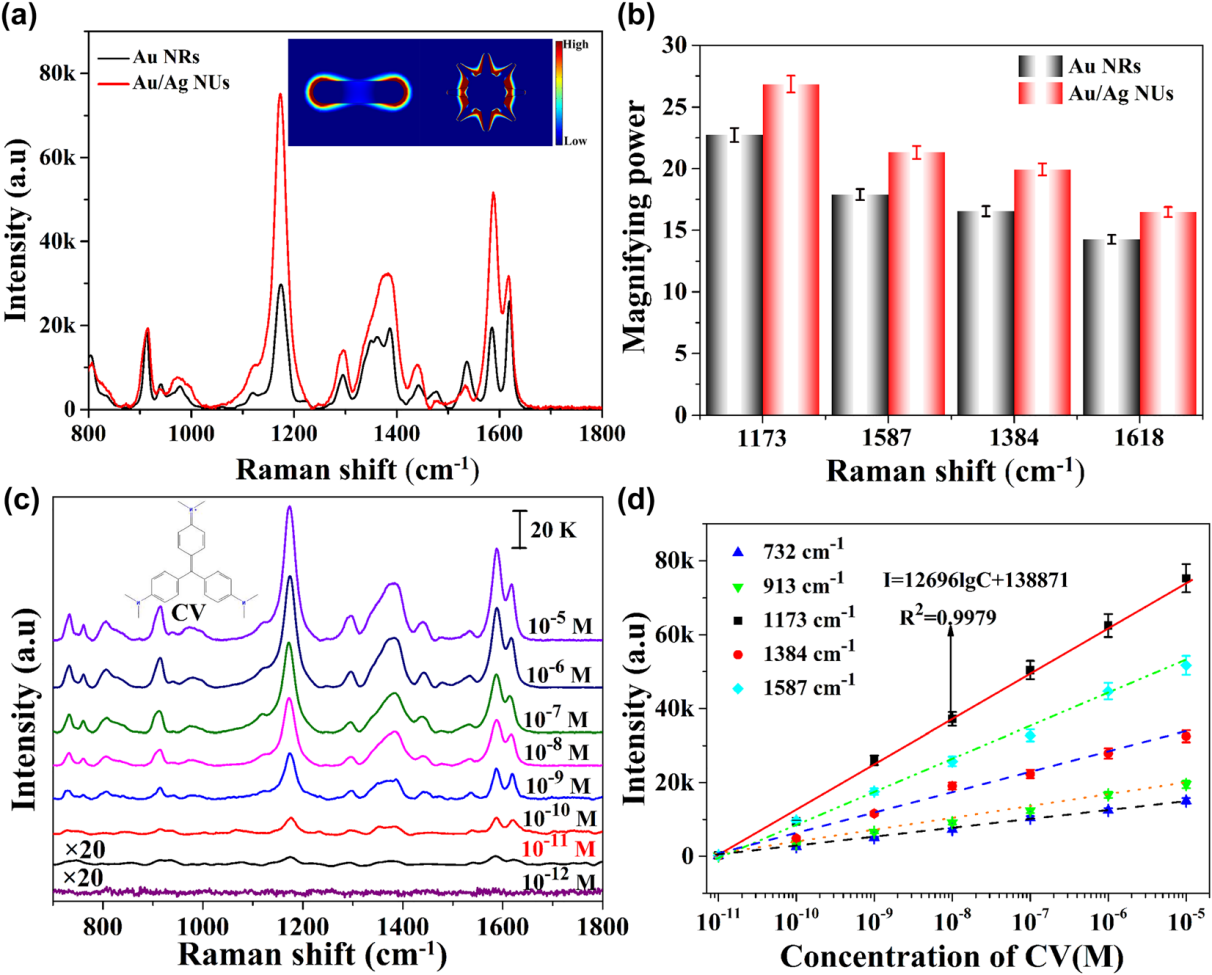
SERS tests of CV molecules in the double laser-induced system. (a) Raman signals of CV (10^−5^ M) in Au/Ag NUs and Au NRs solutions, respectively (Inset shows the FDTD calculations of relative electric field intensities for individual Au NR and Au/Ag NU under 808 nm). (b) The enhanced amplitudes of dual laser systems under Au/Ag NUs and Au NRs substrates (each error bar indicates the standard deviation of 10 different spots). (c) Raman spectra of 10^−5^–10^−12^ M CV via double laser-excited liquid-NIR-SERS. (d) The variations of double laser-triggered Raman intensities at 732, 913, 1173, 1384 and 1587 cm^−1^ versus the CV molecules concentrations in the range of 10^−5^–10^−11^ M (each error bar indicates the standard deviation of 10 different spots).

As we know, traditional SERS activity is highly dependent on the microtopography of SERS nano-substrates, as illustrated in Figure 3. The different Raman enhancements of Au/Ag NUs and Au NRs in the double laser-boosted liquid-NIR-SERS system are also compared, in order to further evaluate the contribution of nano-structure and then offer optimal activity for subsequent researches. Figure 5(a) shows the comparison of liquid-NIR-SERS activities of the two types of different nano-substrates under double laser excitation with the extra laser power of 1.0 W. It can be found that the Raman characteristic bands of CV molecules in the presence of Au/Ag NUs are higher than that of Au NRs. Moreover, based on Au/Ag NUs and Au NRs, we also summarized the enhanced amplitudes of double laser-boosted NIR-SERS in comparison with single laser SERS system, as shown in Figure 5(b). The quantitative comparison clearly shows that the magnifying power of dominating characteristic bands of CV molecules (1173, 1384, 1587 and 1618 cm^−1^) in the presence of Au/Ag NUs are all larger than that of Au NRs, further confirming that the Au/Ag NUs have better Raman enhancement under double-laser excitation. For instance, the Raman intensity of CV molecules at 1384 cm^−1^ in colloidal Au/Ag NUs increases from ∼1525 a.u for traditional single laser excitation to ∼32,497 a.u for the double-laser irradiation. It exhibits an increase of approximate ∼21 times, while the Raman enhancement increases by about ∼17 times via Au NRs substrates. The comparison of other concentrations and characteristic peaks of CV molecules are also show in detail (Tables S1 and S2).

Taking advantage of the optimal candidates, such as optimal confocal region of double laser beam, suitable extra laser power and active nano-architectures of Au/Ag NUs, the detection limit of CV molecules based on double laser-boosted liquid-NIR-SERS was further evaluated, as shown in Figure 5(c). The typical Raman peaks can be still observed even with lower CV concentration of 10^−11^ M, and there are no any Raman characteristic signals can be found by further decreasing molecular concentration to 10^−12^ M. It implies that the detection limit can be achieved as low as 10^−11^ M. The as-synthesized Au/Ag NUs in double laser-excited liquid-NIR-SERS system has the ability to achieve very low molecular detection. Moreover, to investigate the ability of quantitative detection, the intensity–concentration curves of several characteristic peaks located at 732, 913, 1173, 1384 and 1587 cm^−1^ are illustrated in Figure 5(d). It can be found that these five peak intensities display well linear responses to the changes of the logarithm concentrations of CV molecules. For instance, the good linear relationship between the Raman intensity and the logarithm concentration is the linearly dependent coefficient of *R*^2^ = 0.9979 at 1173 cm^−1^, indicating the accuracy and sensitivity of the system. On the other hand, based on as-prepared Au/Ag NUs, the double laser-boosted NIR-SERS system was also extended to the ultrasensitive detection of TC molecules (Figure S10), evaluating the universality and sensitivity of this strategy. It is mainly related to the universal applicability of versatile plasmonic EM mechanism in comparison with specific CT-induced CM enhancement.

**Figure 6:**
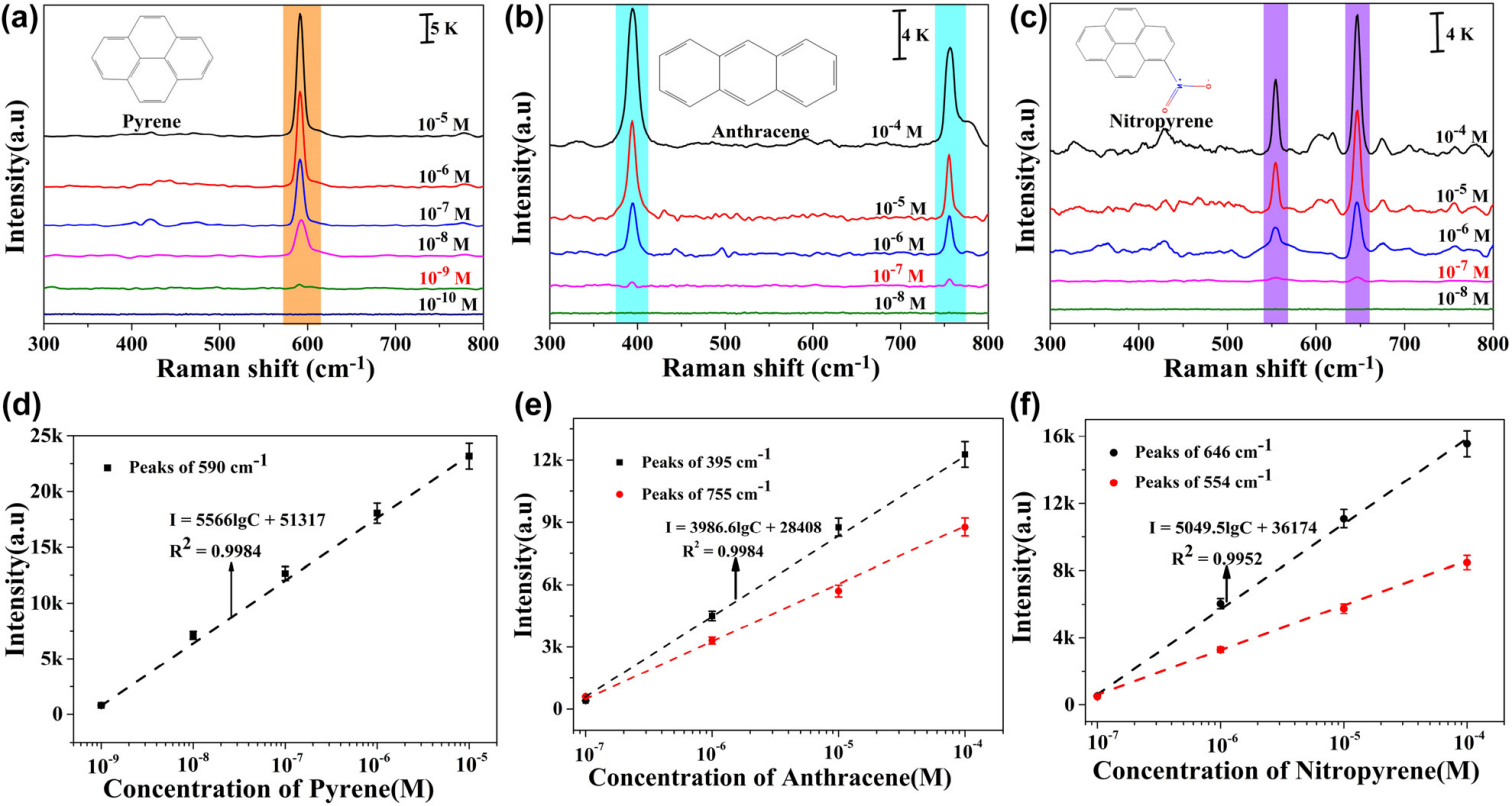
Double laser-boosted liquid-NIR-SERS spectra of (a) pyrene, (b) anthracene, and (c) nitropyrene at different concentrations, in the presence of as-prepared Au/Ag NUs. The insets show the structures of the corresponding probe molecules. The quantitative analyses: (d) the variation of Raman peak intensity at 590 cm^−1^ as a function of pyrene concentration, (e) the variations of Raman peak intensities at 395 cm^−1^ and 755 cm^−1^ as a function of anthracene concentration, and (f) the variations of Raman peak intensities at 646 cm^−1^ and 554 cm^−1^ as a function of nitropyrene concentration, (each error bar indicates the standard deviation of 10 different spots).

Then, the established double laser-boosted liquid NIR-SERS system was practically applied to the ultrasensitive monitoring of three PAHs in solution. In this section, the pyrene, anthracene and nitropyrene molecules were chosen as the model analytes PAHs, which have been frequently found in environmental contamination [56], [57], [58], [59], [60]. The representative pyrene and anthracene molecules are classified as in the U.S. EPA priority PAH list [19]. For instance, Bokam Rajasekhar et al. reported the concentration of anthracene molecules in ground water of 25.4 μg/L [56]; Haroldo S. Dórea et al. determined the concentration of pyrene molecules from the oilfield produced water as 0.9–1.0 μg/L [57]. Meanwhile, the typical nitropyrene molecule is a kind of nitrated derivatives of PAHs, and the corresponding mutagenicity and carcinogenicity may be greater than the original PAHs [61], [62], [63]. In this way, based on as-prepared Au/Ag NUs, the liquid-NIR-SERS tests of 10^−5^–10^−10^ M pyrene, 10^−4^–10^−8^ M anthracene, 10^−4^–10^−8^ M nitropyrene in ethanol/DI solution were performed by double laser excitation, respectively. In order to avoid the interference of ethanol Raman signals in the solvent [64, 65], the Raman tests in the region of 300–800 cm^−1^ were selected in this section. As shown in Figure 6(a–c), the prominent characteristic bands of pyrene, anthracene and nitropyrene molecules are all clearly detected in Raman spectra. In detail, the characteristic band at 590 cm^−1^ is attributed to the ring breathing of pyrene molecules [19, 21]. Moreover, the characteristic bands at 395 and 755 cm^−1^ in Figure 6(b) are related to skeletal deformation and stretching, respectively, demonstrating the presence of anthracene [19, 23]. As shown in Figure 6(c), the Raman peaks of nitropyrene molecules at 554 and 646 cm^−1^ are assigned to ring out of plane and NO_2_ in-plane bending, respectively [19, 66, 67]. More importantly, these dominating characteristic Raman peaks originated from pyrene, anthracene and nitropyrene molecules in Figure 6(a–c) are also clearly distinguishable even the three concentrations decreased as low as 10^−9^, 10^−7^ and 10^−7^ M, respectively. The different NIR-SERS sensitivities toward detection of three kinds of PAHs are related to the different linked functional groups and spatial distributions of their chemical components [19, 21, 23, 24, 28]. Based on the double laser-boosted liquid-NIR-SERS system, the detection limit of PAHs is better than many previous SERS reports obtained by complex functionalized nano-substrates via tedious chemical or physical modifications [21, 23, 24, 28]. To investigate the ability of quantitative detection, the variations of Raman peak intensities of pyrene, anthracene and nitropyrene molecules versus the corresponding concentrations in logarithmical scale are separately shown in Figure 6(d–f). All these plots of Raman peak intensities exhibit well-defined linear responses ranging over wide concentrations of pyrene, anthracene and nitropyrene molecules. Overall, the proposed double laser-boosted NIR-SERS system in the presence of Au/Ag NUs facilitates the ultrasensitive liquid-detections of three different PAHs in solution, which will be beneficial for ultra-trace monitoring in actual water environment.

**Figure 7:**
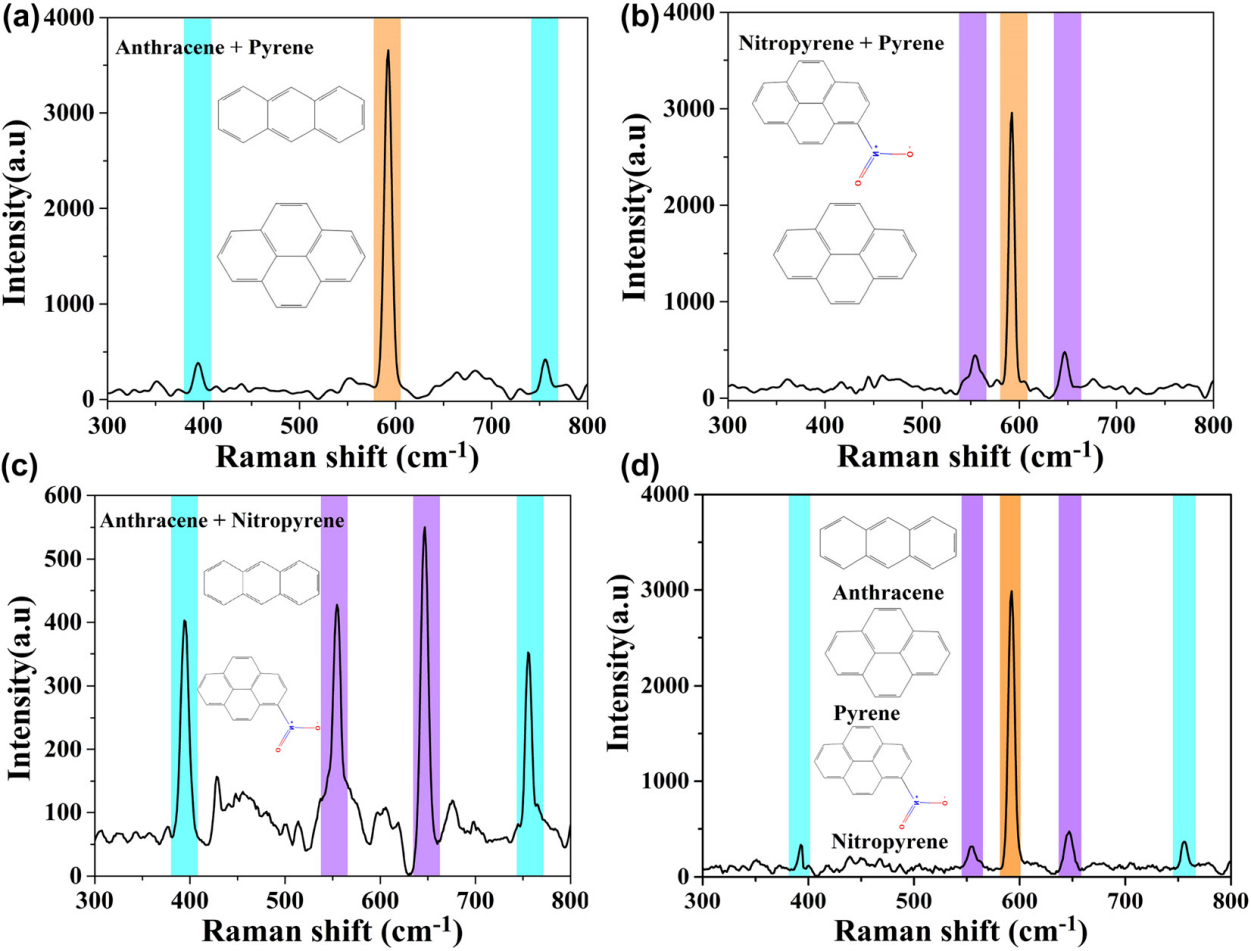
Liquid-NIR-SERS spectra of different mixtures with binary components: anthracene and pyrene (a), nitropyrene and pyrene (b), nitropyrene and anthracene (c), based on the double laser excitation of colloidal Au/Ag NUs. (d) Representative SERS spectra of aqueous ternary mixtures of nitropyrene (purple), anthracene (blue) and pyrene (orange).

As for actual water environment monitoring, besides ultra-low detection, the double laser-boosted liquid-NIR-SERS system should also have good selectively and distinguishability, owing to the real complex environment with the coexistence of two or more pollutant molecules. To provide simultaneous detection of mixture, we mixed these three PAHs molecules in different ratios and analyzed Raman signals of the mixtures in solution by using as-prepared Au/Ag NUs. We designed three groups of mixed solutions including binary components of 10^−6^ M anthracene and 10^−8^ M pyrene, 10^−6^ M nitropyrene and 10^−8^ M pyrene, 10^−6^ M nitropyrene and 10^−6^ M anthracene, as shown in Figure 7(a–c), respectively. In order to maintain the corresponding Raman sensitivity of each component at the similar level in the mixture, the different concentrations of three PAHs were selected in this section, since different Raman enhancements were illustrated toward the separate detections of adopted pyrene, nitropyrene, and anthracene molecules with different values of detection limits. As shown in Figure 7(a–c), every two groups of Raman characteristic signals (marked with corresponding notation) separately originated from two types of PAHs can be clearly identified in each mixed solution. Among them, the Raman peaks at 590 cm^−1^ marked by orange color can be attributed to pyrene molecules. Meanwhile, the peaks at 395 cm^−1^ and 755 cm^−1^ marked by blue color should be belonged to anthracene molecules, while the peaks at 646 cm^−1^ and 554 cm^−1^ marked by purple color should be assigned to nitropyrene molecules. Then, we further applied the liquid-NIR-SERS system to the mixed detection of three PAHs molecules (10^−8^ M pyrene, 10^−6^ M anthracene and 10^−6^ M nitropyrene). As shown in Figure 7(d), the prominent peaks of these three molecules are all clearly detected in NIR-SERS spectra. It can provide much enriched independent “molecular fingerprint” information, confirming that the double laser-boosted liquid-NIR-SERS system in the presence of Au/Ag NUs have excellent SERS spectroscopic distinguishability in this work.

**Figure 8:**
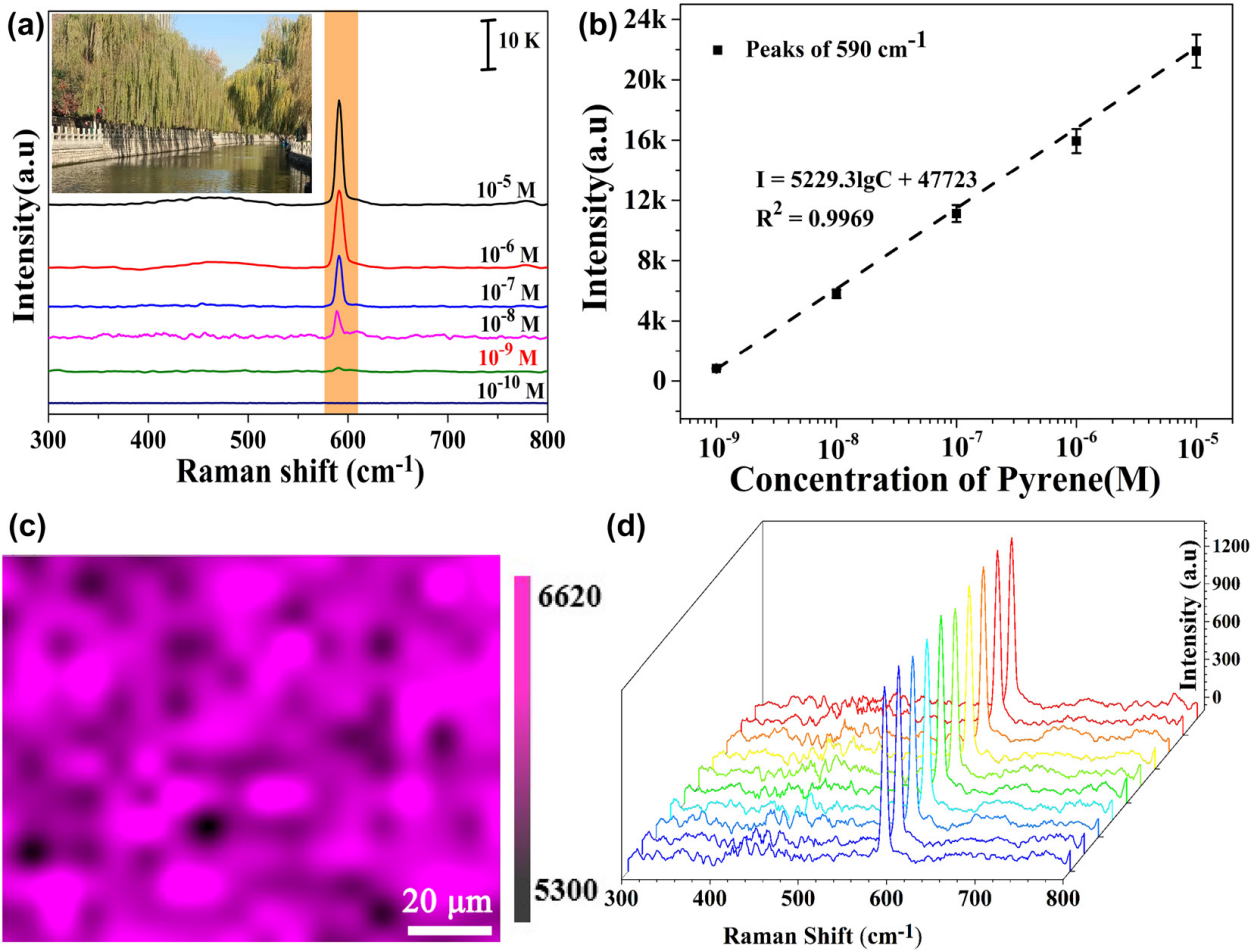
The practical detections of pyrene molecules in real-world scenarios based on double laser-boosted liquid-NIR-SERS. (a) The Raman spectra of pyrene molecules with different concentrations added into lake water solutions. (b) The variations of Raman peak intensity at 590 cm^−1^ versus the pyrene molecules concentrations in the range of 10^−5^–10^−9^ M (each error bar indicates the standard deviation of 10 different spots). (c) The spatial mapping of Raman peak intensity of pyrene molecules (10^−8^ M) at 590 cm^−1^ performed at 252 random spots on Au/Ag NUs-based nano-substrates. (d) The Raman spectra of 10^−9^ M pyrene molecules from 10 different batches of Au/Ag NUs.

Finally, in order to verify the practical applicability of the established double laser-boosted liquid-NIR-SERS system in the actual water environment, the ultrasensitive assessments of different amount of pyrene molecules added in lake water samples were performed in this work. The six groups of lake water samples were filtered and centrifuged before use, and then pyrene molecules were separately added into each solution at different concentrations ranging from 10^−5^ to 10^−10^ M. As shown in Figure 8(a), the Raman characteristic peaks of pyrene molecules at 590 cm^−1^ are successfully detected in the five groups of lake water samples. The detection limit for pyrene molecules can be reached at 10^−9^ M, achieving the MCL detection of PAHs in drinking water. It implies that the established system has great application potential in real water environment. In order to further evaluate the corresponding liquid-NIR-SERS sensitivity, the variation of Raman peak intensity at 590 cm^−1^ versus the pyrene concentrations (logarithmical scale) is shown in Figure 8(b). It reveals an unambiguous linear response over the detection range, which can be expressed by the formula: *I* = 5229.3lgC + 47,723 (*R*^2^ = 0.9969). The results show that some impurities in the lake water do not interfere with the Raman sensitivity of detecting pyrene molecules in this double laser-boosted liquid-NIR-SERS system. The recovery of pyrene molecules was also illustrated by adding known concentrations of pyrene to lake water using standard addition methods [68]. On the basis of the obtained Raman peak intensities, the recovery rates (Table S3) for pyrene molecules were calculated to be 108.31, 97.86 and 103.57%, respectively, in the range of 90–110%, indicating the established strategy can quantify pyrene molecules with high accuracy in lake water [69]. Additionally, in order to verify liquid-NIR-SERS spatial stability of Au/Ag NUs in lake water environment, the spatial mappings (100 × 120 μm) of Raman intensity of pyrene molecules at 590 cm^−1^ is shown in Figure 8(c). It further demonstrates the well-defined homogeneous Raman signal distribution of pyrene molecules in the presence of colloidal Au/Ag NUs. Moreover, the Raman spectra recorded from 10 different batches of Au/Ag NUs-based nano-products are also illustrated in Figure 8(d). It can be seen that the quantitative NIR-SERS measurements can be well repeated on different batches of samples. In addition, the corresponding relative standard deviation (RSD) result of Raman peak intensity at 590 cm^−1^ is calculated about 2.06%, indicating the good reproducibility of double laser-excited liquid-NIR-SERS performed via colloidal Au/Ag NUs. Taken together, the present work exhibits high-performance liquid-NIR-SERS with activity, sensitivity, uniformity, practicability and reproducibility, giving rise to the practical ultrasensitive precise assessment of toxic PAHs in real water environment.

## 4 Conclusions

In summary, we have demonstrated the versatile double laser-boosted liquid-NIR-SERS by introducing an extra 808 nm laser beam into the routine 785 nm laser excitation of Au/Ag NUs in solution. The additional EM field formed around Au/Ag NUs can be promoted by increasing 808 nm external laser power (0–1.0 W), providing another significant contribution for further dramatically promoting liquid-NIR-SERS activity. The optimal candidate enables Raman peak intensities of CV molecules to be dramatically enhanced in solution, reaching approximate ∼34-fold higher than the single 785 nm SERS apparatus. In this way, the detection limit of TC molecules in liquid condition can be down to 10^−9^ M at nanomolar level. Moreover, the unique double laser-boosted NIR-SERS system can realize the ultra-trace liquid detections of nonadsorptive PAHs such as 10^−9^ M pyrene, 10^−7^ M anthracene and 10^−7^ M nitropyrene molecules, respectively. The simultaneous NIR-SERS analyses of these three PAHs show the capabilities of this system for the multiplex detection. Moreover, the practical applicability is successfully verified by ultrasensitive precise assessment of pyrene (10^−5^ M–10^−9^ M) molecules in actual lake water samples. Based on colloidal Au/Ag NUs, the established unique liquid-NIR-SERS is highlighted in the term of well activity, sensitivity, uniformity, distinguishability reproducibility and practicability. These findings are favorable for ultrasensitive and accurate assessment of nonadsorptive pollutants in real-world scenarios.

## Supplementary Material

Supplementary Material Details

## References

[1] Eremina O. E., Sergeeva E. A., Ferree M. V., Shekhovtsova T. N., Goodilin E. A., Veselova I. A. (2020). Dual-Purpose SERS sensor for selective determination of polycyclic aromatic compounds via electron donor–acceptor traps. *ACS Sens.*.

[2] Xue Z., Zheng X., Yu W., Li A., Li S., Wang Y. (2021). Research progress in detection technology of polycyclic aromatic hydrocarbons. *J. Electrochem. Soc.*.

[3] Qazi F., Shahsavari E., Prawer S., Ball A. S., Tomljenovic-Hanic S. (2021). Detection and identification of polyaromatic hydrocarbons (PAHs) contamination in soil using intrinsic fluorescence. *Environ. Pollut.*.

[4] Zhang D., Hao R., Zhang L., You H., Fang J. (2020). Ratiometric sensing of polycyclic aromatic hydrocarbons using capturing ligand functionalized mesoporous au nanoparticles as a surface-enhanced Raman scattering substrate. *Langmuir*.

[5] Jia S., Li D., Fodjo E. K. (2016). Simultaneous preconcentration and ultrasensitive on-site SERS detection of polycyclic aromatic hydrocarbons in seawater using hexanethiol-modified silver decorated graphene nanomaterials. *Anal. Methods*.

[6] Diduch M., Polkowska Ż., Namieśnik J. (2013). Factors affecting the quality of bottled water. *J. Expo. Sci. Environ. Epidemiol.*.

[7] Ratola N., Lacorte S., Barceló D., Alves A. (2009). Microwave-assisted extraction and ultrasonic extraction to determine polycyclic aromatic hydrocarbons in needles and bark of Pinus pinaster Ait. and Pinus pinea L. by GC–MS. *Talanta*.

[8] Wang X. T., Zhou Y., Hu B. P., Fu R., Cheng H. X. (2019). Biomonitoring of polycyclic aromatic hydrocarbons and synthetic musk compounds with Masson pine (Pinus massoniana L.) needles in Shanghai, China. *Environ. Pollut.*.

[9] Premnath N., Mohanrasu K., Rao R. G. R. (2021). A crucial review on polycyclic aromatic Hydrocarbons-Environmental occurrence and strategies for microbial degradation. *Chemosphere*.

[10] Nieva-Cano M. J., Rubio-Barroso S., Santos-Delgado M. J. (2001). Determination of PAH in food samples by HPLC with fluorimetric detection following sonication extraction without sample clean-up. *Analyst*.

[11] Jiang B., Liang Y., Xu C., Zhang J., Hu M., Shi Q. (2014). Polycyclic aromatic hydrocarbons (PAHs) in ambient aerosols from Beijing: characterization of low volatile PAHs by positive-ion atmospheric pressure photoionization (APPI) coupled with Fourier transform ion cyclotron resonance. *Environ. Sci. Technol.*.

[12] Vandergrift G. W., Monaghan J., Krogh E. T., Gill C. G. (2018). Direct analysis of polyaromatic hydrocarbons in soil and aqueous samples using condensed phase membrane introduction tandem mass spectrometry with low-energy liquid electron ionization. *Anal. Chem.*.

[13] Zhang G. L., Zhang M., Shi Q. (2021). In situ construction of COF-based paper serving as a plasmonic substrate for enhanced PSI-MS detection of polycyclic aromatic hydrocarbons. *ACS Appl. Mater. Interfaces*.

[14] Langer J., Jimenez de Aberasturi D., Aizpurua J. (2019). Present and future of surface-enhanced Raman scattering. *ACS Nano*.

[15] Xu K., Zhou R., Takei K., Hong M. (2019). Toward flexible surface-enhanced Raman scattering (SERS) sensors for point-of-care diagnostics. *Adv. Sci.*.

[16] Pilot R., Signorini R., Durante C., Orian L., Bhamidipati M., Fabris L. (2019). A review on surface-enhanced Raman scattering. *Biosensors*.

[17] Bell S. E., Charron G., Cortés E. (2020). Towards reliable and quantitative surface-enhanced Raman scattering (SERS): from key parameters to good analytical practice. *Angew. Chem. Int. Ed.*.

[18] Du J., Jing C. (2011). Preparation of thiol modified Fe3O4@ Ag magnetic SERS probe for PAHs detection and identification. *J. Phys. Chem. C*.

[19] Montes-García V., Gómez-González B., Martínez-Solís D. (2017). Pillar [5] arene-based supramolecular plasmonic thin films for label-free, quantitative and multiplex SERS detection. *ACS Appl. Mater. Interfaces*.

[20] Su M., Wang C., Wang T., Jiang Y., Xu Y., Liu H. (2020). Breaking the affinity limit with dual-phase-accessible hotspot for ultrahigh Raman scattering of nonadsorptive molecules. *Anal. Chem.*.

[21] Gu H. X., Hu K., Li D. W., Long Y. T. (2016). SERS detection of polycyclic aromatic hydrocarbons using a bare gold nanoparticles coupled film system. *Analyst*.

[22] Mueller M., Tebbe M., Andreeva D. V. (2012). Large-area organization of pNIPAM-coated nanostars as SERS platforms for polycyclic aromatic hydrocarbons sensing in gas phase. *Langmuir*.

[23] Wang X., Hao W., Zhang H. (2015). Analysis of polycyclic aromatic hydrocarbons in water with gold nanoparticles decorated hydrophobic porous polymer as surface-enhanced Raman spectroscopy substrate. *Spectrochim. Acta A*.

[24] Montes‐García V., Fernández‐López C., Gómez B. (2014). Pillar [5] arene-mediated synthesis of gold nanoparticles: size control and sensing capabilities. *Chem. Eur J.*.

[25] Li D., Cao X., Zhang Q. (2019). Facile in situ synthesis of core–shell MOF@ Ag nanoparticle composites on screen-printed electrodes for ultrasensitive SERS detection of polycyclic aromatic hydrocarbons. *J. Mater. Chem. A*.

[26] Lu H., Zhu L., Zhang C., Chen K., Cui Y. (2018). Mixing assisted “hot spots” occupying SERS strategy for highly sensitive in situ study. *Anal. Chem.*.

[27] Zhang R., Lai Y., Zhan J. (2021). Enhancing the activity of silver nanowire membranes by electrochemical cyclic voltammetry as highly sensitive flexible SERS substrate for on-site analysis. *Nanomaterials*.

[28] Zhang M., Zhang X., Qu B., Zhan J. (2017). Portable kit for high-throughput analysis of polycyclic aromatic hydrocarbons using surface enhanced Raman scattering after dispersive liquid-liquid microextraction. *Talanta*.

[29] Liu C., Zhang X., Li L. (2015). Silver nanoparticle aggregates on metal fibers for solid phase microextraction–surface enhanced Raman spectroscopy detection of polycyclic aromatic hydrocarbons. *Analyst*.

[30] Zhang M., Zhang X., Shi Y. E., Liu Z., Zhan J. (2016). Surface enhanced Raman spectroscopy hyphenated with surface microextraction for in-situ detection of polycyclic aromatic hydrocarbons on food contact materials. *Talanta*.

[31] Castro-Grijalba A., Montes-García V., Cordero-Ferradás M. J., Coronado E., Pérez-Juste J., Pastoriza-Santos I. (2020). SERS-based molecularly imprinted plasmonic sensor for highly sensitive PAH detection. *ACS Sens.*.

[32] Ye T., Huang Z., Zhu Z. (2020). Surface-enhanced Raman scattering detection of dibenzothiophene and its derivatives without π acceptor compound using multilayer Ag NPs modified glass fiber paper. *Talanta*.

[33] Qiu X., You X., Chen X. (2017). Development of graphene oxide-wrapped gold nanorods as robust nanoplatform for ultrafast near-infrared SERS bioimaging. *Int. J. Nanomed.*.

[34] Yang Y. T., Hsu I. L., Cheng T. Y. (2019). Off-Resonance SERS nanoprobe-targeted screen of biomarkers for antigens recognition of bladder normal and aggressive cancer cells. *Anal. Chem.*.

[35] Kang H., Jeong S., Jo A. (2018). Ultrasensitive NIR-SERRS probes with multiplexed ratiometric quantification for in vivo antibody leads validation. *Adv. Healthcare Mater.*.

[36] Zhou L., Zhou J., Lai W. (2020). Irreversible accumulated SERS behavior of the molecule-linked silver and silver-doped titanium dioxide hybrid system. *Nat. Commun.*.

[37] Wang P., Lux L., Jin M. (2018). Au/Ag nanobox-based near-infrared surface-enhanced Raman scattering for hydrogen sulfide sensing. *ACS Appl. Bio Mater.*.

[38] Wang H. N., Register J. K., Fales A. M. (2018). Surface-enhanced Raman scattering nanosensors for in vivo detection of nucleic acid targets in a large animal model. *Nano Res.*.

[39] Liu Z., Chen H., Jia Y. (2018). A two-dimensional fingerprint nanoprobe based on black phosphorus for bio-SERS analysis and chemo-photothermal therapy. *Nanoscale*.

[40] Kang H., Jeong S., Park Y. (2013). Near-infrared SERS nanoprobes with plasmonic Au/Ag hollow-shell assemblies for in vivo multiplex detection. *Adv. Funct. Mater.*.

[41] Greeneltch N. G., Davis A. S., Valley N. A. (2012). Near-infrared surface-enhanced Raman spectroscopy (NIR-SERS) for the identification of eosin Y: theoretical calculations and evaluation of two different nanoplasmonic substrates. *J. Phys. Chem. A*.

[42] Murphy C. J., Thompson L. B., Chernak D. J. (2011). Gold nanorod crystal growth: from seed-mediated synthesis to nanoscale sculpting. *Curr. Opin. Colloid Interface Sci.*.

[43] Nikoobakht B., El-Sayed M. A. (2003). Preparation and growth mechanism of gold nanorods (NRs) using seed-mediated growth method. *Chem. Mater.*.

[44] Zhang H., Chen M., Wang D., Xu L., Liu X. (2016). Laser induced fabrication of mono-dispersed Ag 2 S@ Ag nano-particles and their superior adsorption performance for dye removal. *Opt. Mater. Express*.

[45] Xu L., Li S., Zhang H., Wang D., Chen M. (2017). Laser-induced photochemical synthesis of branched Ag@ Au bimetallic nanodendrites as a prominent substrate for surface-enhanced Raman scattering spectroscopy. *Opt. Express*.

[46] Meng M., Fang Z., Zhang C. (2016). Integration of kinetic control and lattice mismatch to synthesize Pd@ AuCu core–shell planar tetrapods with size-dependent optical properties. *Nano Lett.*.

[47] Wang J. L., Ando R. A., Camargo P. H. (2014). Investigating the plasmon-mediated catalytic activity of AgAu nanoparticles as a function of composition: are two metals better than one. *ACS Catal.*.

[48] Tian Y., Zhang H., Xu L., Chen M., Chen F. (2018). Self-assembled monolayers of bimetallic Au/Ag nanospheres with superior surface-enhanced Raman scattering activity for ultra-sensitive triphenylmethane dyes detection. *Opt. Lett.*.

[49] Jia K., Xie J., He X. (2020). Polymeric micro-reactors mediated synthesis and assembly of Ag nanoparticles into cube-like superparticles for SERS application. *Chem. Eng. J.*.

[50] Li X., Zhu J., Wei B. (2016). Hybrid nanostructures of metal/two-dimensional nanomaterials for plasmon-enhanced applications. *Chem. Soc. Rev.*.

[51] Canamares M. V., Chenal C., Birke R. L., Lombardi J. R. (2008). DFT, SERS, and single-molecule SERS of crystal violet. *J. Phys. Chem. C*.

[52] Liang E. J., Ye X. L., Kiefer W. (1997). Surface-enhanced Raman spectroscopy of crystal violet in the presence of halide and halate ions with near-infrared wavelength excitation. *J. Phys. Chem. A*.

[53] Kleinman S. L., Ringe E., Valley N. (2011). Single-molecule surface-enhanced Raman spectroscopy of crystal violet isotopologues: theory and experiment. *J. Am. Chem. Soc.*.

[54] Zhang C., Li Z., Qiu S. (2022). Highly ordered arrays of hat-shaped hierarchical nanostructures with different curvatures for sensitive SERS and plasmon-driven catalysis. *Nanophotonics*.

[55] Mu Y., Liu M., Li J., Zhang X. (2021). Plasmonic hollow fibers with distributed inner-wall hotspots for direct SERS detection of flowing liquids. *Opt. Lett.*.

[56] Rajasekhar B., Nambi I. M., Govindarajan S. K. (2018). Human health risk assessment of ground water contaminated with petroleum PAHs using Monte Carlo simulations: a case study of an Indian metropolitan city. *J. Environ. Manag.*.

[57] Dórea H. S., Bispo J. R., Aragão K. A. (2007). Analysis of BTEX, PAHs and metals in the oilfield produced water in the State of Sergipe, Brazil. *Microchem. J.*.

[58] Zhou J. L., Maskaoui K. (2003). Distribution of polycyclic aromatic hydrocarbons in water and surface sediments from Daya Bay, China. *Environ. Pollut.*.

[59] Beiranvand M., Ghiasvand A. (2020). Design and optimization of the VA-TV-SPME method for ultrasensitive determination of the PAHs in polluted water. *Talanta*.

[60] Manabe Y., Kinouchi T., Wakisaka K., Tahara I., Ohnishi Y. (1984). Mutagenic 1-nitropyrene in wastewater from oil-water separating tanks of gasoline stations and in used crankcase oil. *Environ. Mutagen.*.

[61] Dos Santos R. R., Leal L. D. V., de Lourdes Cardeal Z., Menezes H. C. (2019). Determination of polycyclic aromatic hydrocarbons and their nitrated and oxygenated derivatives in coffee brews using an efficient cold fiber-solid phase microextraction and gas chromatography mass spectrometry method. *J. Chromatogr. A*.

[62] Knecht A. L., Goodale B. C., Truong L. (2013). Comparative developmental toxicity of environmentally relevant oxygenated PAHs. *Toxicol. Appl. Pharmacol.*.

[63] Bandowe B. A. M., Meusel H. (2017). Nitrated polycyclic aromatic hydrocarbons (nitro-PAHs) in the environment–a review. *Sci. Total Environ.*.

[64] Frausto-Reyes C., Medina-Gutiérrez C., Sato-Berrú R., Sahagún L. R. (2005). Qualitative study of ethanol content in tequilas by Raman spectroscopy and principal component analysis. *Spectrochim. Acta A*.

[65] Li F., Men Z., Li S., Wang S., Li Z., Sun C. (2018). Study of hydrogen bonding in ethanol-water binary solutions by Raman spectroscopy. *Spectrochim. Acta A*.

[66] Carrasco-Flores E. A., Clavijo R. E., Campos-Vallette M. M., Aroca R. F. (2004). Vibrational spectra and surface-enhanced vibrational spectra of 1-nitropyrene. *Appl. Spectrosc.*.

[67] Carrasco E. A., Campos-Vallette M., Leyton P. (2003). Study of the interaction of pollutant nitro polycyclic aromatic hydrocarbons with different metallic surfaces by surface-enhanced vibrational spectroscopy (SERS and SEIR). *J. Phys. Chem. A*.

[68] Westley C., Xu Y., Thilaganathan B., Carnell A. J., Turner N. J., Goodacre R. (2017). Absolute quantification of uric acid in human urine using surface enhanced Raman scattering with the standard addition method. *Anal. Chem.*.

[69] Tian Y., Cui Q., Xu L. (2021). Alloyed AuPt nanoframes loaded on h-BN nanosheets as an ingenious ultrasensitive near-infrared photoelectrochemical biosensor for accurate monitoring glucose in human tears. *Biosens. Bioelectron.*.

