# Supplementary material for: Synergistic double laser beam-boosted liquid-NIR-SERS for ultralow detection of non-adsorptive polycyclic aromatic hydrocarbons in lake water
Source: Nanophotonics. 2022 Apr 25;11(12):2875–89. doi: 10.1515/nanoph-2022-0010 (PMC11501877; doi:10.1515/nanoph-2022-0010)
Supplement: Supplementary file 1 — Supplementary Material Details [file j_nanoph-2022-0010_suppl.doc]

Supplementary Information

Synergistic double laser beam-boosted liquid-NIR-SERS for ultralow detection of non-adsorptive polycyclic aromatic hydrocarbons in lake water

*Mengya Zhang^1^, Yue Tian^1^, Anxin Jiao^1^, Hui Ma^1^, Chang Wang^1^, Linqi Zheng^2^, Shuang Li^2*^ and Ming Chen^1*^*

^1^School of Physics, Shandong University, Jinan, 250100, P. R. China,

^2^School of Science, Shandong Jianzhu University, Jinan, 250101, P. R. China

^*^Corresponding author: [chenming@sdu.edu.cn](mailto:chenming@sdu.edu.cn), lishuang@sdjzu.edu.cn


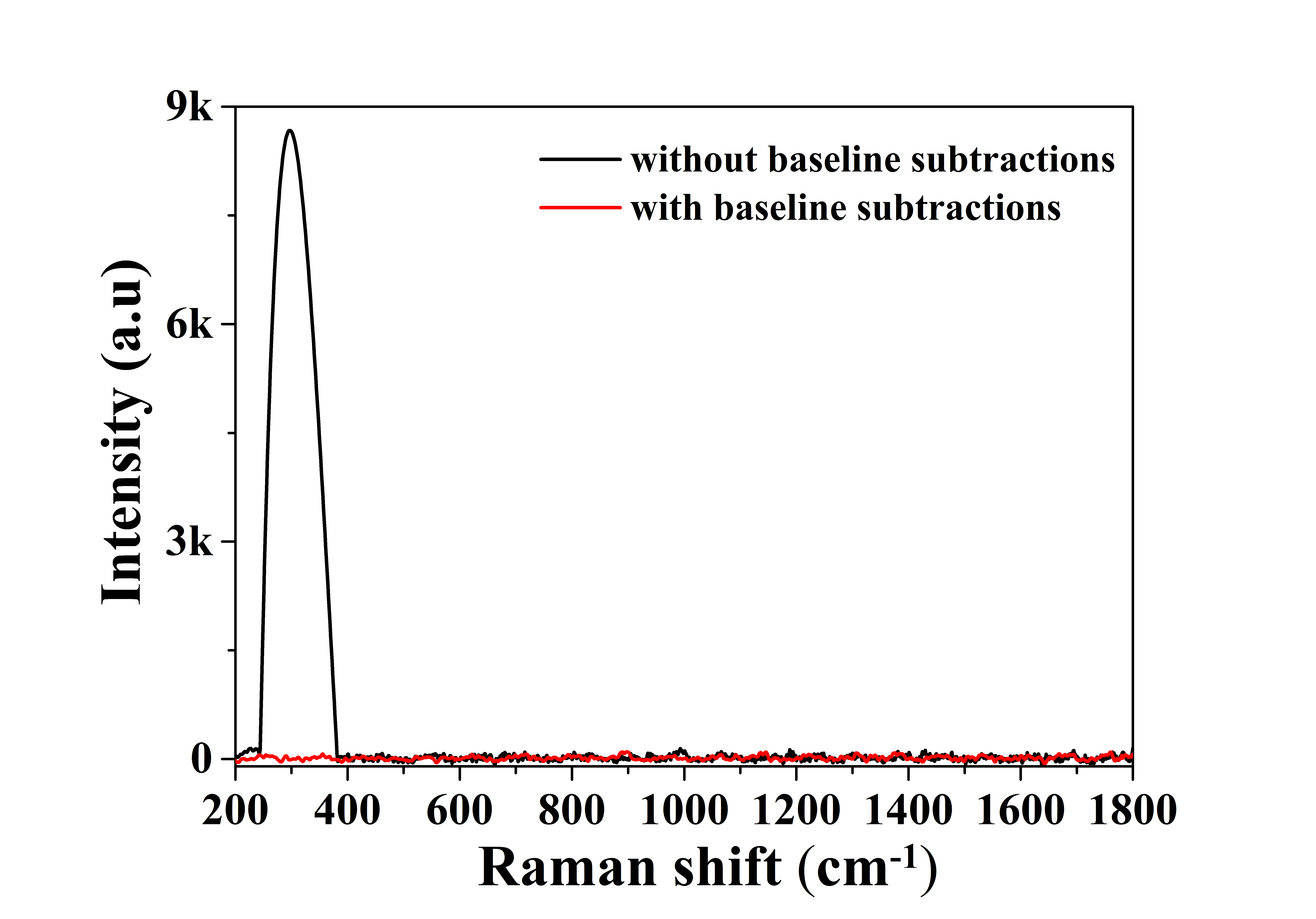


Figure S1: The Raman spectra with and without baseline subtractions in the presence of Au/Ag NUs after the introduction of 808 nm laser.


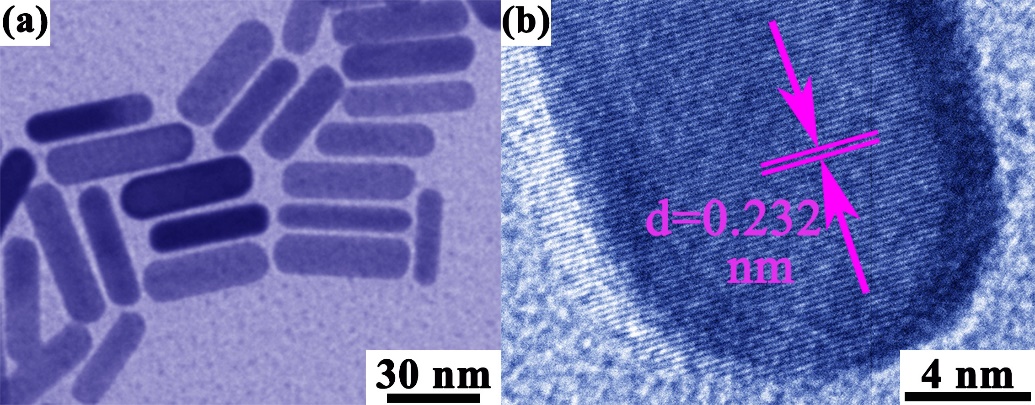


Figure S**2**: (a) The TEM image and (b) representative HRTEM image of Au NRs.


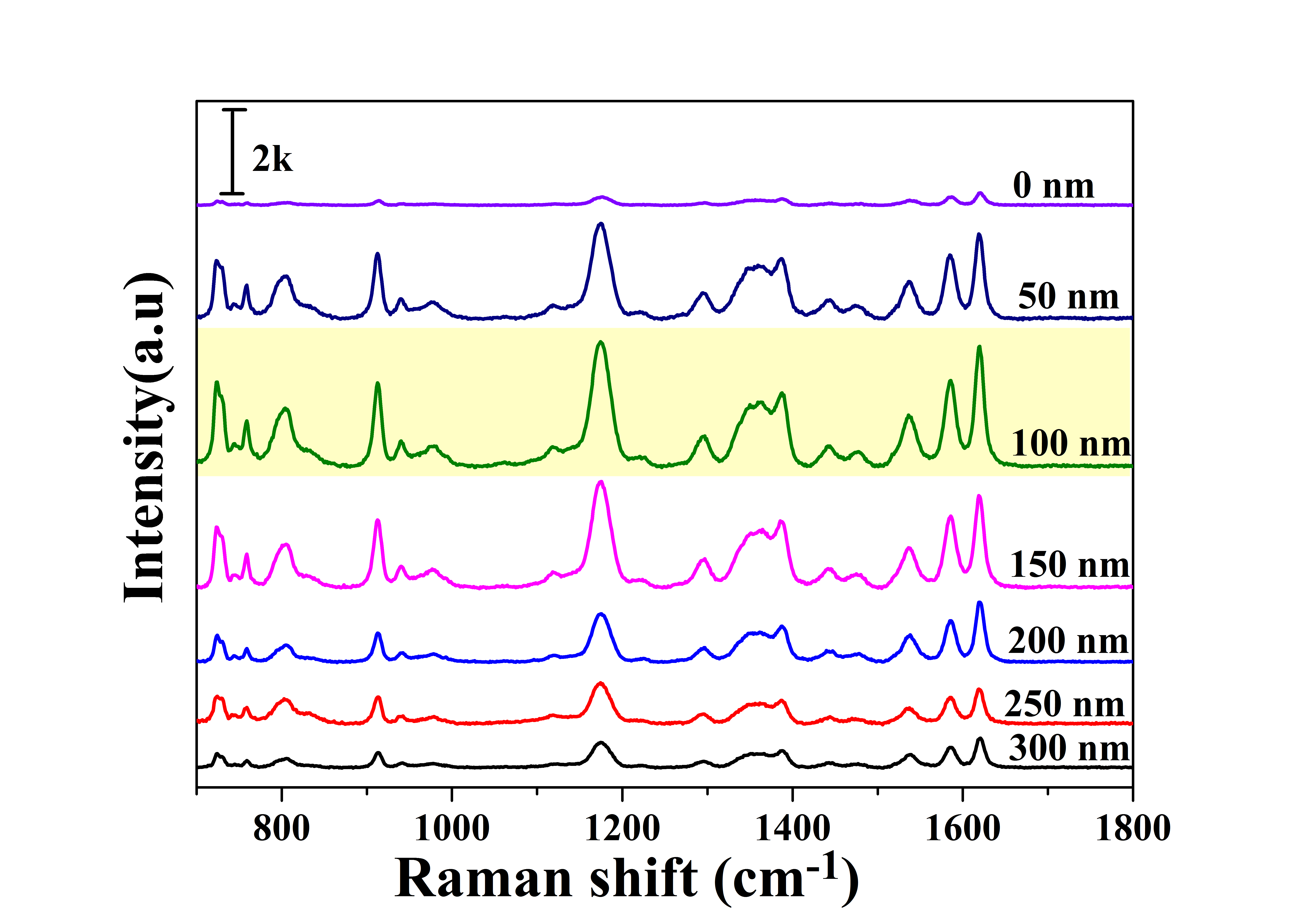


**Figure S3:** Raman spectra of 10^−5^ M CV molecules in solution at different depths along the vertical *Z-*direction. The solution surface was defined as the zero point at vertical direction.


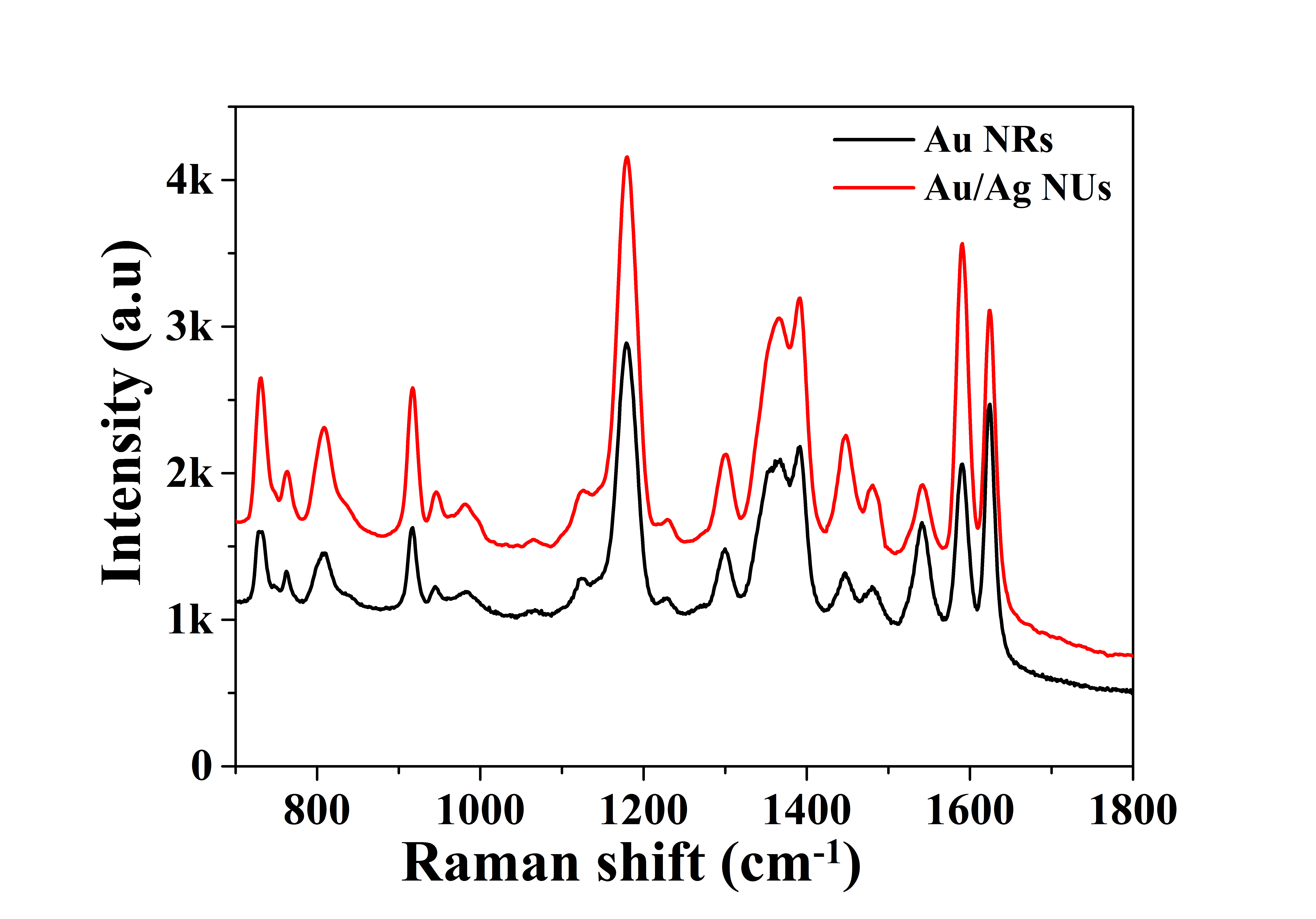


**Figure S4:** The original Raman spectra of 10^−5^ M CV molecules separately performed in the presence of Au/Ag NUs and Au NRs without baseline subtractions.


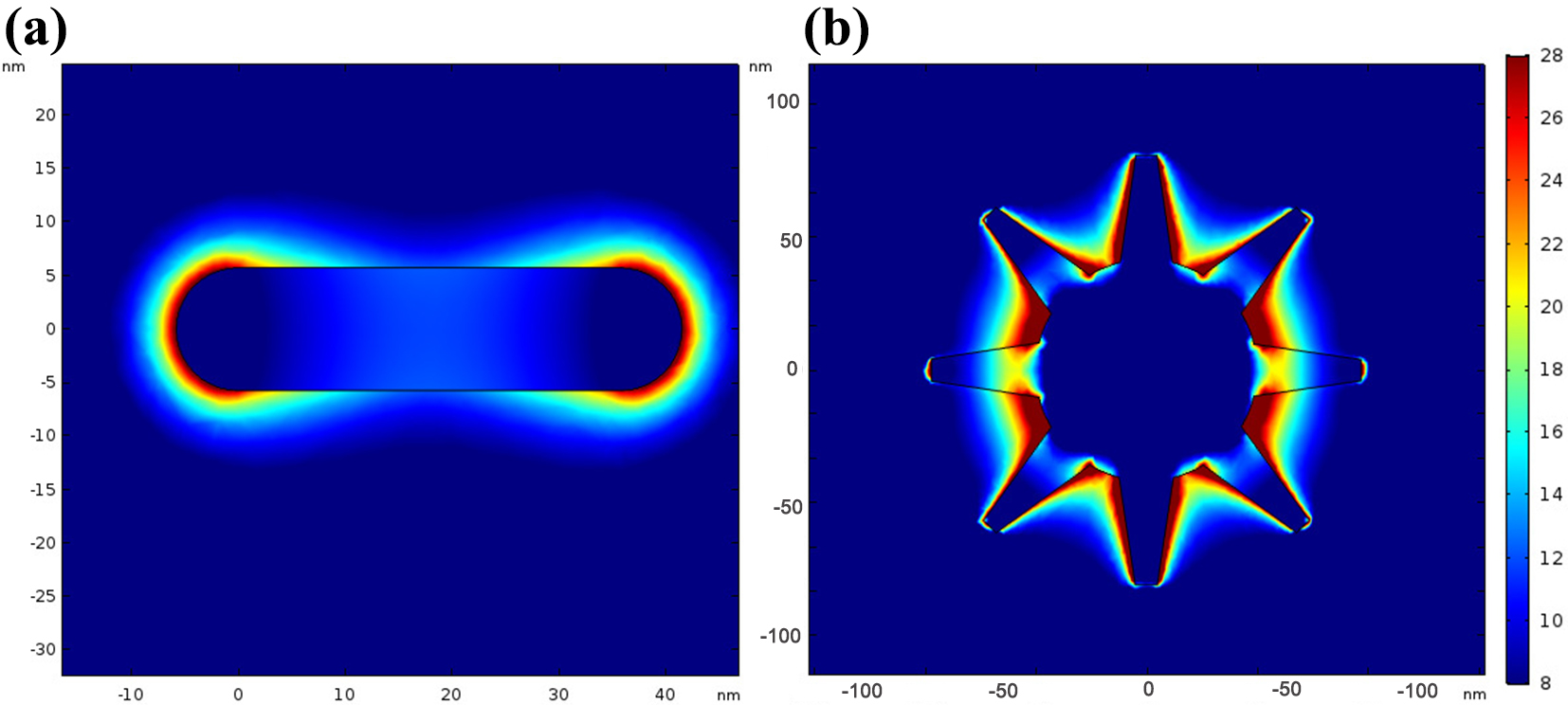


Figure S**5**: (a−b) FDTD calculations of relative electric field intensities for individual Au NR and Au/Ag NU, the incidence wavelength in FDTD simulations was selected at 785 nm.


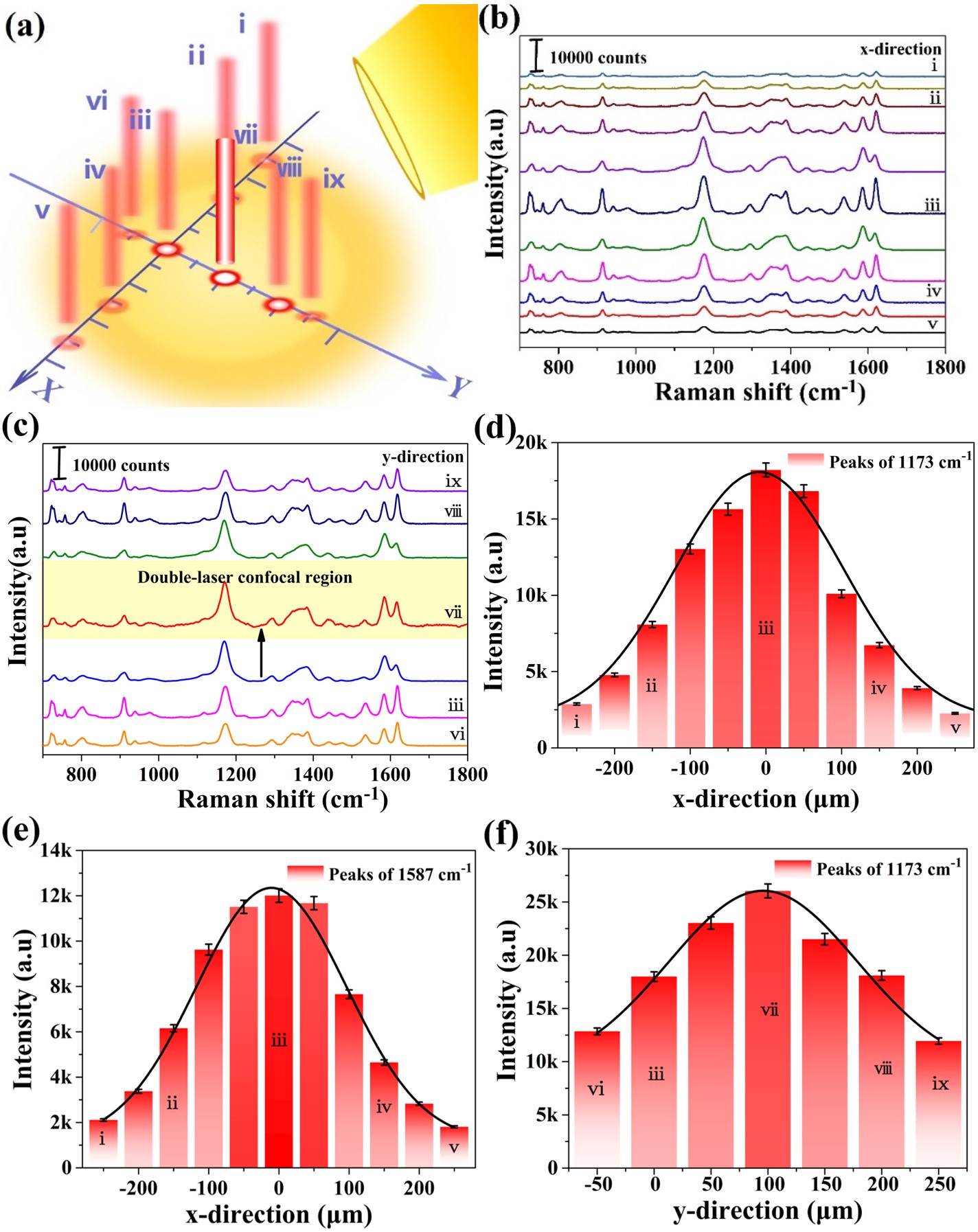


Figure S**6**: (a) The schematic diagram of dual beam focusing process. (b−c) Scanning Raman spectra of 10^−9^ M CV molecules versus relative *X-* and *Y-*positions of 808 nm laser beam with respect to 785 nm focused point. (d−e) Raman intensity of characteristic peak at 1173 cm^−1^ and 1587 cm^−1^ versus x-offset, black line shows the trend of the signal changes, (each error bar indicates the standard deviation of ten different spots). (f) Raman intensity of characteristic peak at 1173 cm^−1^ versus y-offset, black line shows the trend of the signal changes, (each error bar indicates the standard deviation of ten different spots).


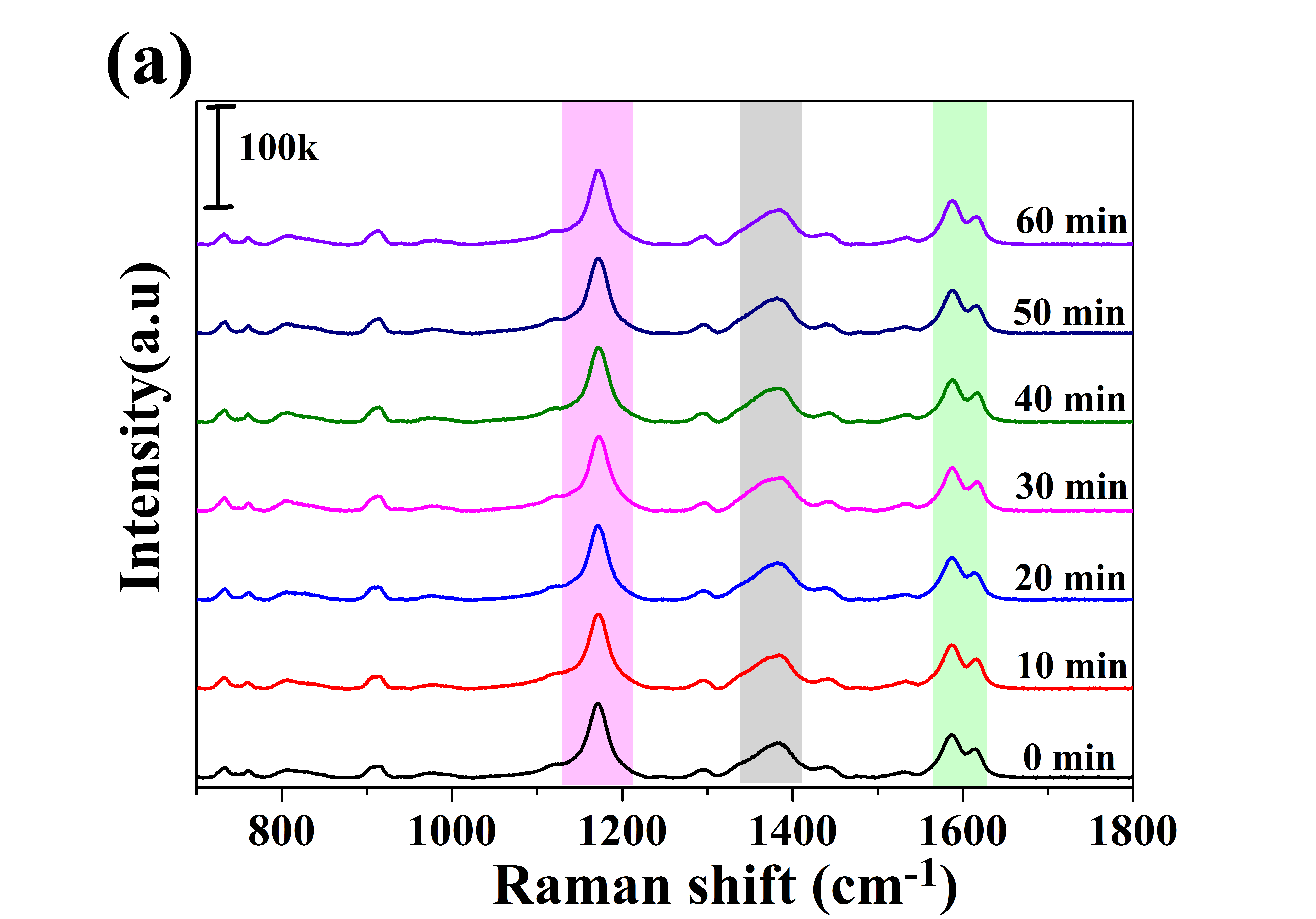

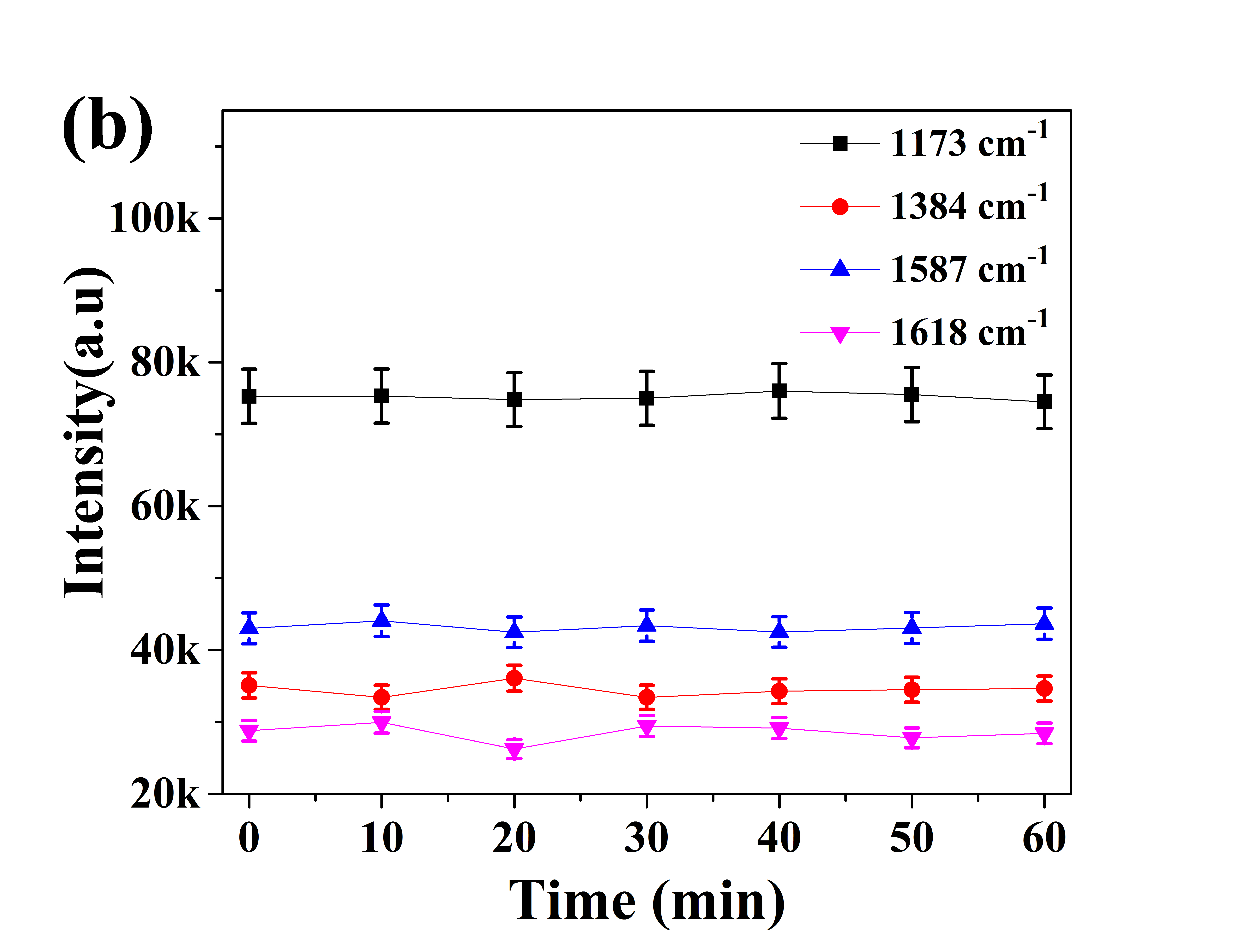


Figure S**7**: (a) The Raman spectra of CV (10^−5^ M) on Au/Ag NUs substrates irradiated by 785 nm and 808 nm (1.0 W) lasers at different delay times (0-60 min). (b) The curves of intensity at peaks 1173, 1384, 1587, 1618 cm^−1^ with different irradiation times, (each error bar indicates the standard deviation of ten different spots).


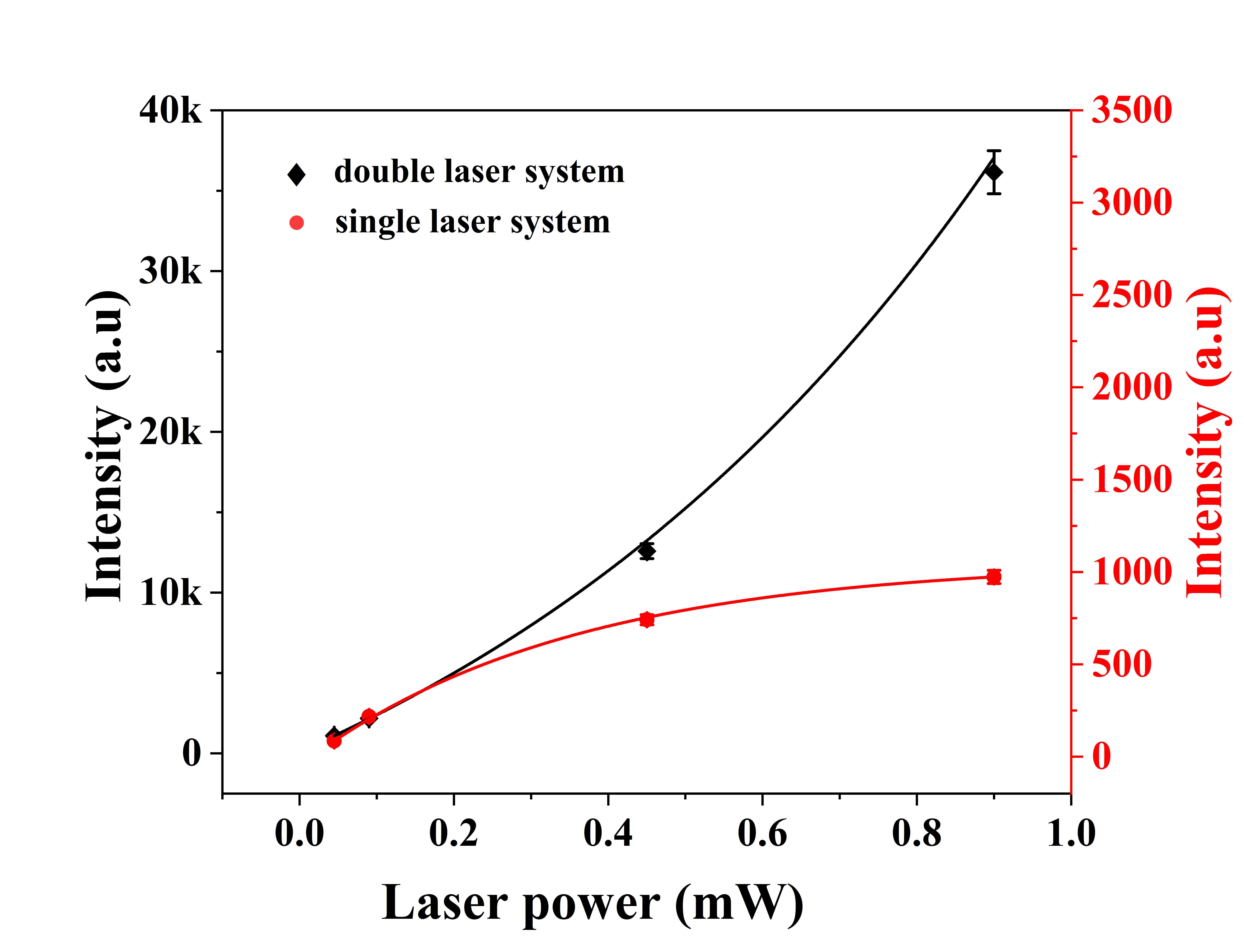


Figure S**8:** The point-to-point statistics of the variation of Raman peak at 1173 cm^-1^ in the single laser system (red points and line) and double laser system (black points and line) with changing the power of the 785 nm Raman excitation light source in each system (each error bar indicates the standard deviation of ten different spots).


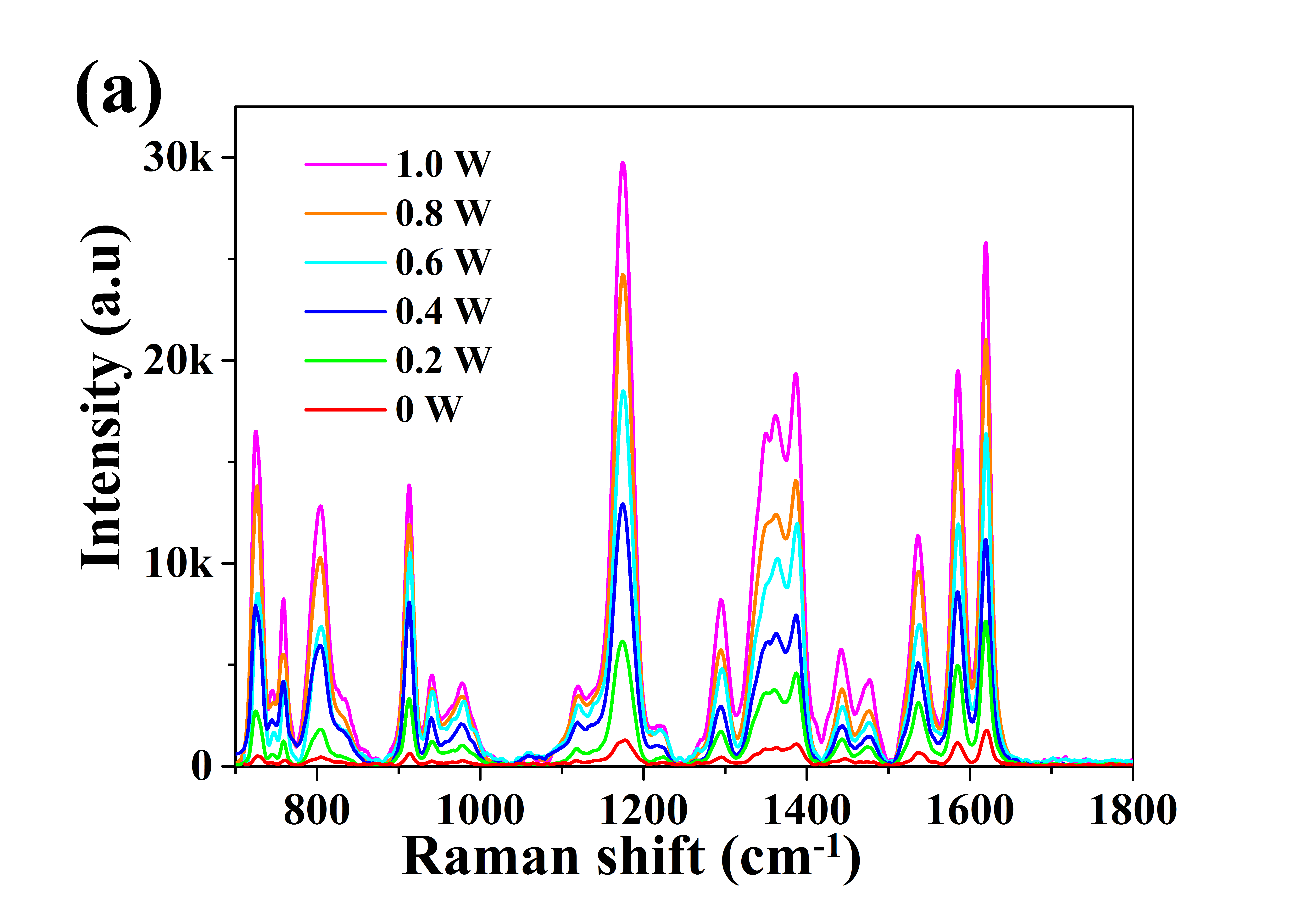

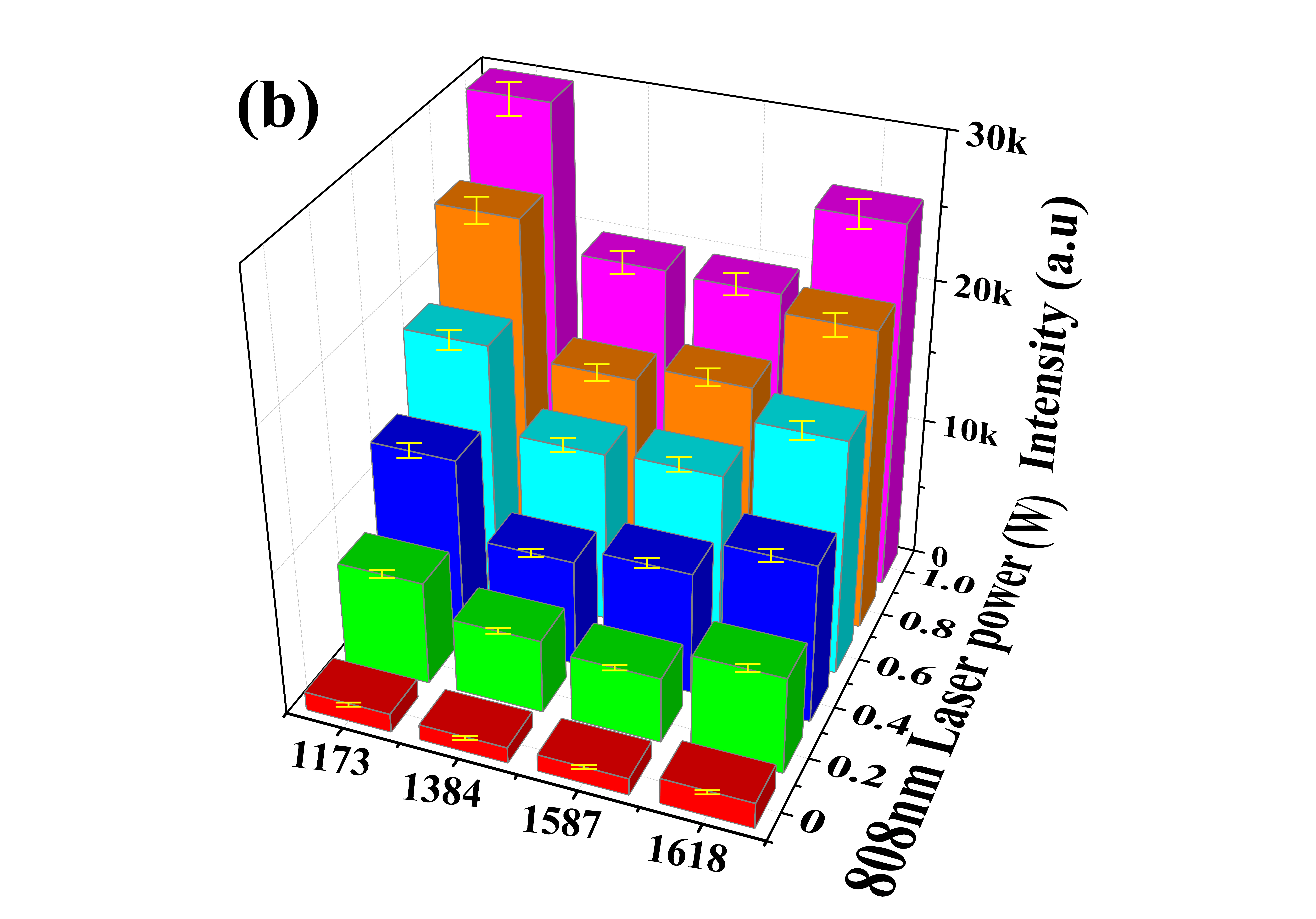


Figure S**9**: (a) Raman signals of CV (10^−5^ M) in Au NRs solution with different extra 808nm laser powers. (b) The variation trends of the main four Raman peaks intensities of CV molecules versus different extra 808 nm laser power (0~1.0 W) (each error bar indicates the standard deviation of ten different spots).


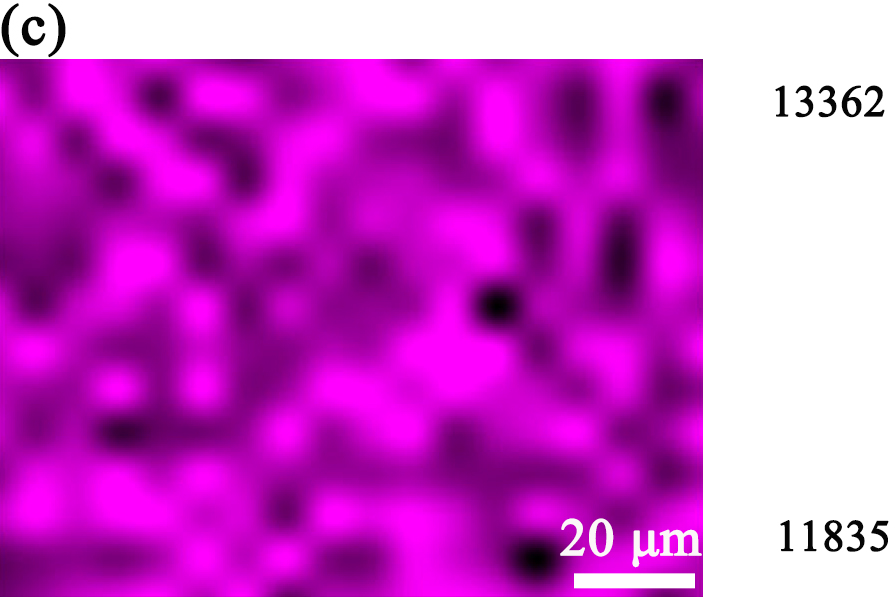

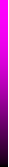

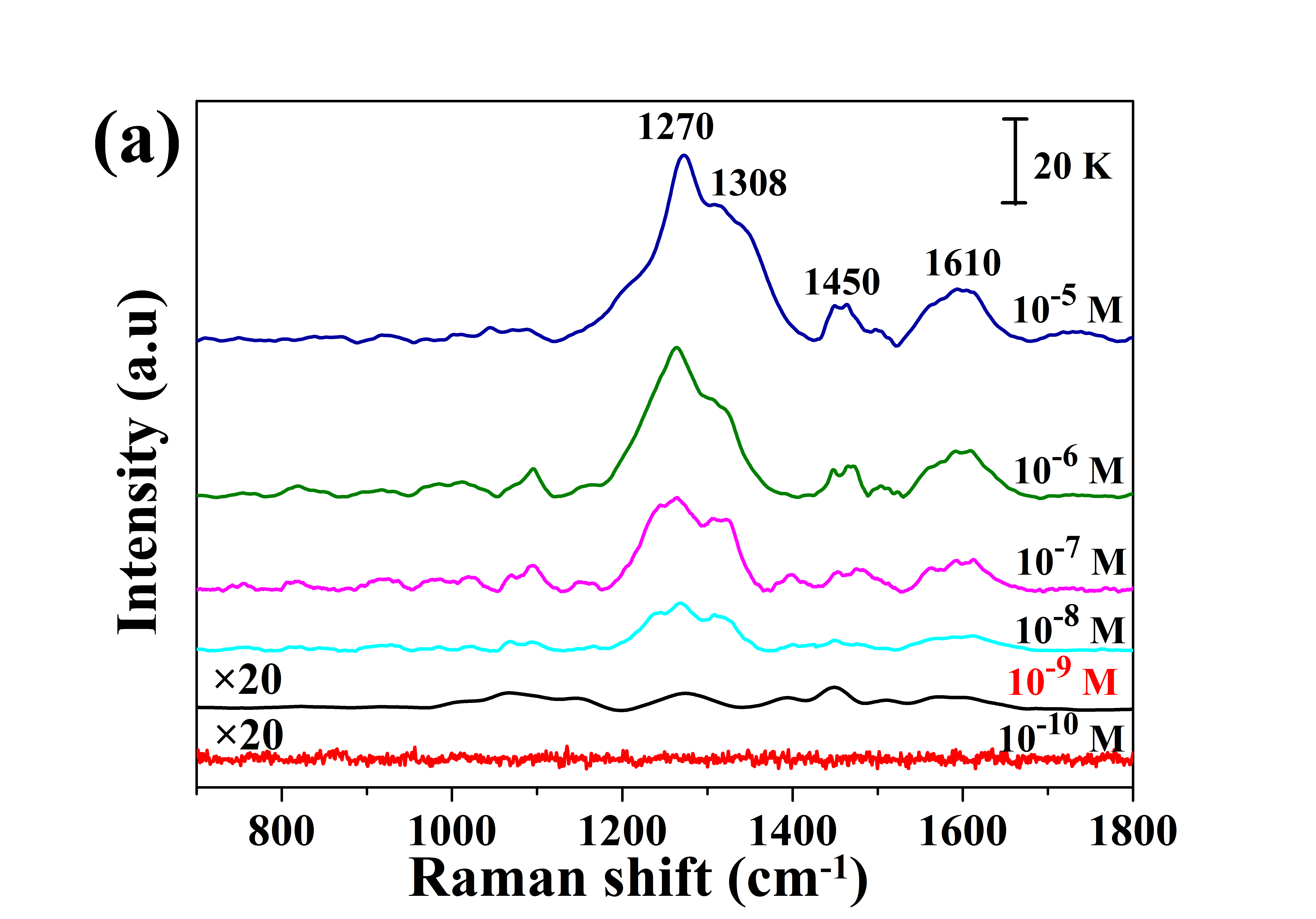

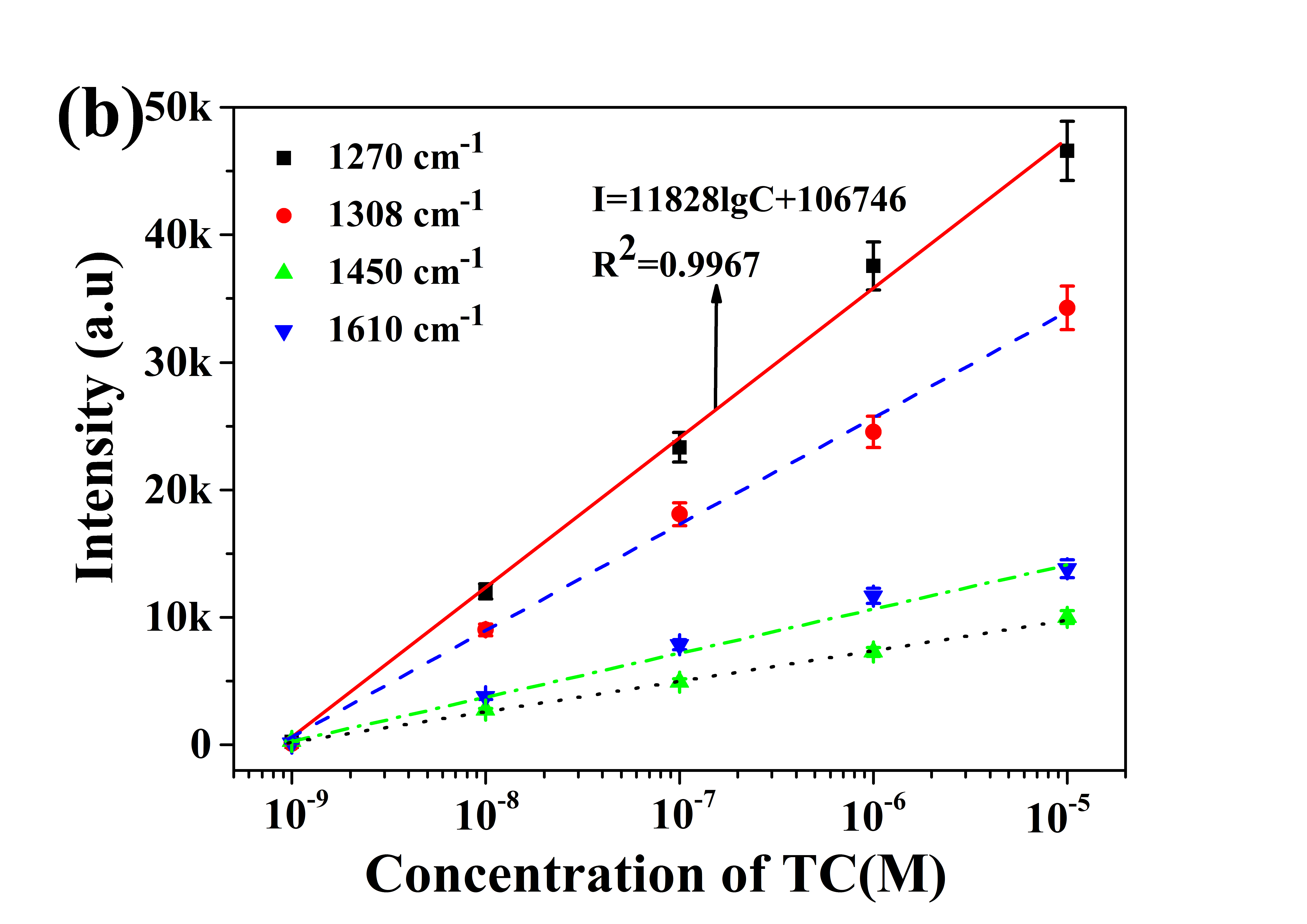


**11934**

Figure S**10**: (a) Based on the optimal NIR-SERS, the Raman spectra of TC molecules with different concentrations in the presence of Au/Ag NUs. (b) The variations of double laser-boosted NIR-SERS intensities at 1270, 1308, 1450 and 1610 cm^−1^ versus the TC molecules concentrations in the range of 10^−5^~10^−9^ M, (each error bar indicates the standard deviation of ten different spots). (c) The spatial mapping of Raman peak intensity of TC molecules (10^−8^ M) at 1270 cm^-1^ performed at 252 random spots on Au/Ag NUs-based nano-substrates.

**Table S1:** The magnification factors of CV Raman signals on Au/Ag NUs with different concentrations under double laser excitation (The power of 808 nm laser is 1.0 W) (each standard deviation comes from 10 different spots).

| **Concentration** | **1173 cm^−1^** | **1384 cm^−1^** | **1587 cm^−1^** | **1618 cm^−1^** |
| --- | --- | --- | --- | --- |
| 10^−5^ M | 27.04 ± 0.83 | 21.31 ± 0.55 | 19.93 ± 0.73 | 16.47 ± 0.27 |
| 10^−6^ M | 28.00 ± 0.94 | 22.44 ± 0.57 | 21.88 ± 0.54 | 17.54 ± 0.45 |
| 10^−7^ M  10^−8^ M  10^−9^ M  10^−10^ M | 28.59 ± 0.68  30.27 ± 0.82  34.48 ± 0.98  33.42 ± 0.77 | 21.40 ± 0.35  27.67 ± 0.79  22.83 ± 0.75  21.94 ± 0.53 | 21.65 ± 0.41  24.96 ± 0.54  27.87 ± 0.80  28.10 ± 0.92 | 16.55 ± 0.36  19.06 ± 0.81  22.98 ± 0.71  28.65 ± 0.89 |

**Table S2:** The magnification factors of CV Raman signals on Au NRs with different concentrations under double laser excitation (The power of 808 nm laser is 1.0 W) (each standard deviation comes from 10 different spots).

| **Concentration** | **1173 cm^−1^** | **1384 cm^−1^** | **1587 cm^−1^** | **1618 cm^−1^** |
| --- | --- | --- | --- | --- |
| 10^−5^ M | 22.73 ± 0.87 | 17.89 ± 0.36 | 16.54 ± 0.44 | 14.25 ± 0.37 |
| 10^−6^ M  10^−7^ M  10^−8^ M | 14.81 ± 0.93  18.74 ± 0.85  17.13 ± 0.64 | 18.47 ±0.96  17.68 ± 0.83  23.28 ± 0.91 | 18.55 ± 0.89  20.78 ± 0.51  18.10 ± 0.50 | 19.23 ±0.66  19.54 ± 0.68  18.73 ± 0.86 |

**Table S3:** Recovery rates of pyrene molecules in laker water samples, as determined by the standard addition method (each RSD comes from 10 different spots).

| **Samples** | **Added(nM)** | **Found(nM)** | **Recovery** | **RSD** |
| --- | --- | --- | --- | --- |
| 1  2  3 | 10  100  1000 | 10.83  103.57  978.60 | 108.31%  103.57%  97.86% | 2.92%  5.91%  3.86% |
